# *Quo Vadis* Venomics? A Roadmap to Neglected Venomous Invertebrates

**DOI:** 10.3390/toxins6123488

**Published:** 2014-12-19

**Authors:** Bjoern Marcus von Reumont, Lahcen I. Campbell, Ronald A. Jenner

**Affiliations:** Department of Life Sciences, the Natural History Museum, Cromwell Road, SW7 5BD London, UK

**Keywords:** venoms, Remipedia, Glyceridae, Asilidae, Tabanidae, Sciomyzidae, Chilopoda, Pseudoscorpiones, Nemertea, *Acanthaster*

## Abstract

Venomics research is being revolutionized by the increased use of sensitive -omics techniques to identify venom toxins and their transcripts in both well studied and neglected venomous taxa. The study of neglected venomous taxa is necessary both for understanding the full diversity of venom systems that have evolved in the animal kingdom, and to robustly answer fundamental questions about the biology and evolution of venoms without the distorting effect that can result from the current bias introduced by some heavily studied taxa. In this review we draw the outlines of a roadmap into the diversity of poorly studied and understood venomous and putatively venomous invertebrates, which together represent tens of thousands of unique venoms. The main groups we discuss are crustaceans, flies, centipedes, non-spider and non-scorpion arachnids, annelids, molluscs, platyhelminths, nemerteans, and echinoderms. We review what is known about the morphology of the venom systems in these groups, the composition of their venoms, and the bioactivities of the venoms to provide researchers with an entry into a large and scattered literature. We conclude with a short discussion of some important methodological aspects that have come to light with the recent use of new -omics techniques in the study of venoms.

## 1. Introduction

Animal venoms are complex proteinaceous cocktails that have evolved independently in as many as two dozen lineages to serve predation, defense, communication, and competition [1,2,3,4]. Venoms are typically delivered via a wound, and their specific activities are principally determined by their mix of proteins and peptides, which are individually referred to as toxins. However, the biology and evolution of animals’ venoms is very unevenly understood.

Venomics research is being revolutionized by the use of highly sensitive and high-throughput transcriptomic and proteomic techniques, as well as the increasing availability of genomic resources. The impact of these technological advances is noticeable across the discipline. They enable unexpected new insights into the biology and evolution of some of the most intensely studied and best understood venom systems, such as cone snails and snakes [5,6], but at the same time they are dramatically accelerating research into neglected or even completely unstudied venomous taxa, such as centipedes, the platypus, polychaetes and remipede crustaceans [7,8,9].

The ability of the -omics technologies to bring neglected taxa within the purview of venomics is especially important if we want to understand the true diversity of venom systems in the animal kingdom, and if we want our generalizations about the biology and evolution of venoms not to be overly biased by the insights garnered from only the best studied taxa, such as cone snails, snakes, spiders, and scorpions. Three recent examples illustrate how the application of new -omics techniques to neglected taxa has yielded insights at odds with our general understanding of venoms. First, the current paradigm that venom toxin genes generally result from gene duplication followed by recruitment to venom glands is not supported by insights derived from the platypus [9]. Gene duplication played a role in the origin of only 16 out of 107 platypus genes homologous to known toxin genes. Second, von Reumont *et al.* [7] discovered that toxin gene expression in the venom glands of remipede crustaceans is dominated by enzymes, with only a single suspected neurotoxin. This is sharply at odds with toxin gene expression in the venom glands of the three main groups of venomous predatory arthropods: centipedes, spiders, and scorpions. The venoms of these animals are dominated by the expression of a great diversity of neurotoxic peptides. Third, Undheim *et al.* [10] discovered that the venom glands of scolopendromorph centipedes express multidomain toxin transcripts. Among venomous invertebrates this is very rare, and only known to occur in coleoid cephalopods and some arthropods [10]. It appears that these toxin genes are under strong negative selection, which is in contrast to the majority of predatory toxins, the evolution of which is reigned by positive selection.

Given the importance of studying neglected taxa to generate such new insights, and given that the vast majority of neglected venomous taxa are invertebrates, our paper aims to provide the outlines of a roadmap to neglected venomous invertebrates for future venomics studies. We provide brief reviews of what is known about the venom systems of these taxa, including the general morphology of their venom apparatus, the composition of their venoms, and the bioactivities of their suspected venoms. This should help interested researchers enter the relevant literature.

## 2. Arthropoda

Arthropoda is a group of invertebrates that comprises roughly 75%–85% of all known species on earth [11,12]. Their evolution traces back more than 520 Mio years, and yet many questions concerning arthropod relationships remain [11,13], in particular for the four traditional extant euarthropod groups (chelicerates, myriapods, crustaceans, insects). For instance, one remaining challenge is to understand how insects conquered land, after splitting from a common ancestor shared perhaps exclusively with remipede crustaceans [11,14]. Crustaceans, insects, myriapods and chelicerates occur in almost all known habitats and play important ecological roles [11]. In these taxa tens of thousands of venomous species have evolved that possess an enormous diversity of complex toxin arsenals [3,4]. Venomous species are especially common among hymenopteran insects, chelicerates, and centipedes. Some arthropod groups even exclusively comprise venomous species [15], such as spiders and scorpions. It is therefore unsurprising that arthropod venoms are recognized as one of the greatest resources of biologically active molecules in nature [12,16,17].

Hymenopteran insects, in particular ants and bees, have since ancient times been the subject of traditional folk practices in medicine and cultural rituals. The therapeutic use of honey-bee venom even dates back to the time of ancient Egypt, Greece and the Roman Empire. Hippocrates (460–377 BC), for example, describes for the first time the use of bee stings and bee venom to treat arthritis. The potential of antimicrobial and viral applications of bee venom components has recently been described [18]. By establishing automated and efficient proteomic methods in the late 1990s, and phylogenomic analyses since 2008 [19,20,21,22,23] early studies on species relevant to humans were expanded. In particular, venom composition and medically and/or economically important components like venom allergen or immune suppressor proteins were described and studied in more detail for groups like bees and parasitoid wasps, and other arthropod species that followed and adapted to human civilization like scorpions and some spiders. Especially the parasitoid wasp *Nasonia vitripennis* and its venom are exceptionally well studied, which is also linked to the recent genome sequencing project of this species. New insights show, for instance, that its venom has anti-inflammatory action [24].

Yet, although quite a few arthropod venom toxins have been isolated and characterized both structurally and functionally, we generally know much less about the toxin composition of arthropod venom cocktails, even in arthropod groups that have long been studied, like hymenopterans. This is also reflected in the relatively small number of transcriptomic data sets based on hymenopteran venom gland tissue that are available today (see Table S1). That paradoxical situation is now changing with the emergence of increasingly affordable -omics technology. Further, new allergological approaches utilize these technologies, for example, in the “component resolved diagnosis”, in which specific antigenes and immuno responses are tested. This approach to molecular diagnosis from the late 1990s is applied in particular to hymenopteran venoms and has recently been discussed [25,26,27].

However, here we will focus on hitherto neglected and/or understudied venomous arthropod taxa, such as centipedes, some groups of flies, and remipede crustaceans, groups to which the new technologies have begun to create access to.

### 2.1. Remipedes, the First Venomous Crustaceans

The first transcriptomic profile of a crustacean venom was only recently published [7]. Before then crustaceans were the only major traditional arthropod group for which no venomous species were known. Of course examples of poisonous crustaceans, mostly crabs or lobsters, have long been known, but in these cases poison compounds derive from microorganisms or plant material ingested by the crustaceans [28,29], which can lead to food poisoning when these are in turn ingested by humans.

**Figure 1:**
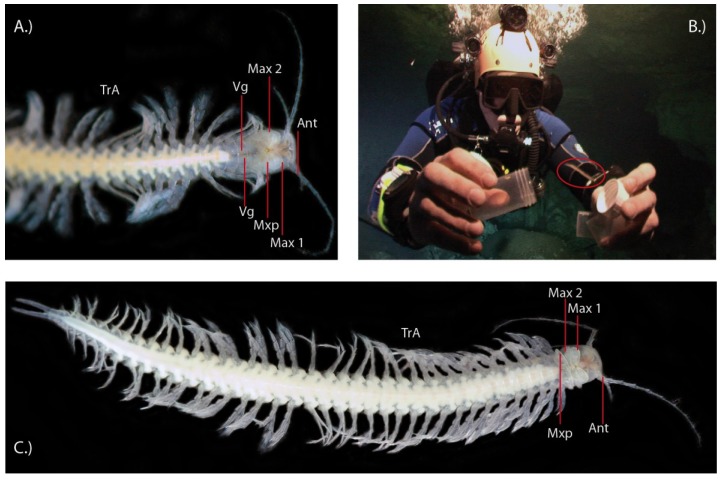
The first venomous crustacean, *Xibalbanus tulumensis*. (**A**) The cephalon and part of the trunk is shown from the ventral side. TrA = trunk appendages, Vg = venom gland, Mxp = maxilliped, Max 2 = maxilla 2, Max1 = maxilla 1 (= maxillule), Ant = antenna (antennula); (**B**) An individual (marked in red circle) is caught in the Mexican anchialine cave system “Cenote Crustacea” by BMvR; (**C**) Habitus of a specimen, showing its remarkably convergent bodyplan to centipedes with homonomously segmented trunk and similar trunk appendages (TrA).

The first venomous crustacean, *Xibalbanus tulumensis* (formerly *Speleonectes tulumensis*, Yager (1987); see Hoenemann *et al.* [30]) belongs to the crustacean class Remipedia, which consists only of cave dwelling, blind, pigment-less species (Figure 1). Remipedes live as obligate stygobionts in the saltwater parts of anchialine underwater cave systems that are generally rather nutrient poor. After their relatively recent description in 1981 (Yager) [31], remipedes were assumed to represent an ancient crustacean lineage that had split from the remaining crustacean early on, and which had retained a mostly primitive body plan, with a long, homonomously segmented trunk furnished with a series of similar biramous swimming legs [32]. However, recent molecular and neuroanatomical studies suggest that remipedes are instead a rather derived crustacean group that is closely related to Hexapoda [14,33,34,35].

**Figure 2:**
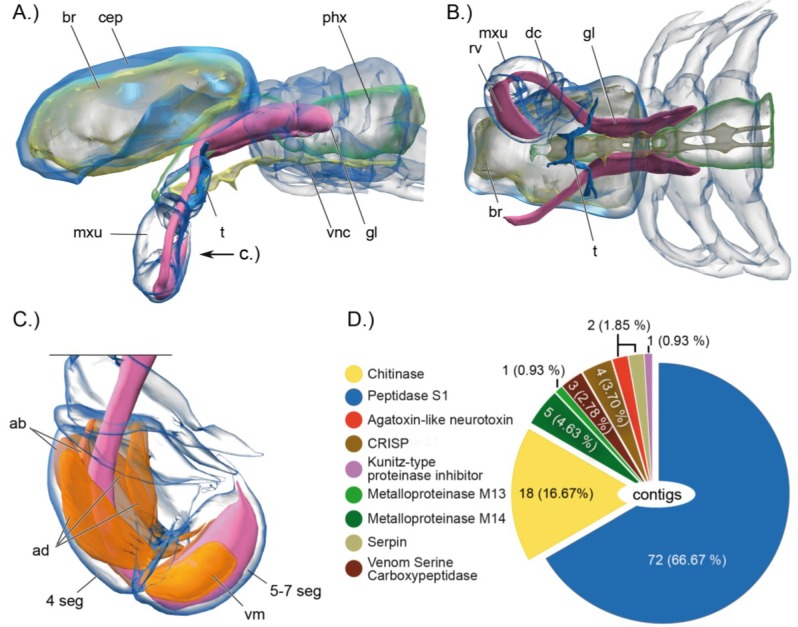
Synchrotron-based computer tomographic reconstruction of the cephalothorax and the venom delivery system of the remipede *Xibalbanus tulumensis* in lateral view (anterior to the left), (**A**), and in ventral view (**B**), and the muscle system that facilitates venom injection by the maxillule (**C**); (**D**) shows the composition of the cocktail of toxin gene transcripts expressed in the venom glands. Abbreviations: 4 seg = 4th segments of maxillule, ab = abductor muscles, ad = adductor muscles br = brain, cep = cephalothorax, phx = pharynx, mxu = maxillula, t = tegument, vnc = ventral nerv cord, gl = venom gland, dc = venom duct, rv = venom reservoir, vm = ventral apodemal muscle.

A decade and a half after their description, two studies suggested that remipedes could possibly be venomous and that the tips of their fang-like maxillulae are connected via a venom reservoir with a pair of venom glands that are located in the first segments of the cephalothorax [36,37]. The recent paper of von Reumont *et al.* [7] provides the most detailed picture yet of the functional morphology of the remipede venom apparatus. The synchrotron scan-based 3D reconstructions clearly show a complex venom delivery system that is able to inject venom in a controlled manner (Figure 2A–C). Remipedes have a pair of venom glands in the first three thoracic segments. Each gland leads via a venom duct to a venom reservoir located in the maxillules, which are powerful appendages used for grabbing and stabbing prey. The maxillulae end in a sharp tip where the venom reservoir opens via a subterminal pore [7,37]. The study of von Reumont *et al.* [7] also tested Van der Ham and Felgenhauer’s [35,37] hypothesis for how remipede venom might work. Van der Ham and Felgenhauer found that homogenized remipede venom glands have phenoloxidase activity, and that injection of an enzyme with phenoloxidase activity (laccase) could harm or kill shrimp, but only when a substrate of the enzyme (4-methylcatechol) was injected into the shrimp as well. From these findings Van der Ham and Felgenhauer concluded that phenoloxidase activity might produce venomous effects in remipede prey. They further proposed that the source of phenoloxidase activity in remipede venom was hemocyanin, the individual subunits of which are known to have phenoloxidase activity. They diagnosed the presence of hemocyanin in the venom glands on the basis of the presence of hemocyanin-like electron dense components in electron micrographs of the remipede venom glands. They therefore posited that remipedes would have to inject at least three components into prey: hemocyanin, an unknown substance that could dissociate hemocyanin into its enzymatically active subunits, and an unknown phenoloxidase substrate. However, transcriptomic profiling of toxin gene expression in remipede venom glands does not support this rather convoluted hypothesis.

Remipede venom glands express no transcripts for phenoloxidase, and only 15 reads of a single hemocyanin transcript are expressed [7]. Interestingly, however, it was shown in 2009 that remipedes do express three hemocyanin subunits [33]. Von Reumont *et al.* therefore concluded that the phenoloxidase activity found in the experiments of Van der Ham and Felgenhauer probably results from contamination with hemolymph, in which hemocyanin is present and probably functions as an oxygen carrier.

It was shown that more than 80% of the toxin gene transcripts expressed in the venom glands of remipedes represent chitinase and peptidase S1 sequences. However, the venom glands also express transcripts coding for a putative neurotoxin very similar to one known only from agelenid funnel web spiders (Figure 2D). This agatoxin-like neurotoxin is known to induce spastic paralysis of insect prey [38].

The transcriptomic profile suggests that the composition of remipede venom allows them to adopt an “arachnoid” way of feeding [39], which draws further support from morphology and field observations. Remipedes have an unusually muscular esophagus and have been seen to ingest the internal tissue of prey crustaceans, after which they released the empty cuticular husk of the prey [39,40]. The expressed enzymes could break up chitinous cuticular structures, while the proteases could macerate the prey’s tissue culminating in an easily ingested liquid meal. As blind obligate stygobionts that live in nutrient poor underwater cave systems it is obviously very adaptive if prey specimens can be paralyzed immediately to minimize risk of losing the catch. The highly expressed transcripts for the agatoxin-like neurotoxin precursor corroborates this hypothesis. Because of their inaccessible habitats observations of feeding remipedes in the field are rare. Only one photograph of a remipede that caught a shrimp has been published, and the general method of prey capture in the wild remains unknown, although some laboratory observations have been made [41,42]. Observations in captivity show that remipedes also eat dead prey and may also feed on particles [41,42]. A mixed mode of feeding could have an adaptive advantage in the nutrient poor habitats in which they live.

### 2.2. Other Neglected Putatively Venomous Crustaceans

Observations as old as the mid-18th century suggest that there are other crustaceans that may have venom glands and are putatively venomous, see for instance in Møller [43]. Two belong to parasitic, economically important crustacean groups, the branchiurans (fish lice) and the siphonostomatoid copepods (sea lice). Interestingly, both possess a very similar morphology, which is probably convergent due to their parasitic mode of life on fish. Another taxon is Caprellidae (skeleton shrimps), which are amphipods and have recently been in the spotlight for being invasive crustaceans [44,45,46].

#### 2.2.1. Fish Lice (Branchiura)

Branchiurans are ectoparasitic crustaceans that occur mostly on freshwater fish and consequently were already known for a long time as a pest species before the group was described in 1864 (Thorell), see [43]. The position of this parasitic group within crustaceans and their internal phylogenetic relationships remain unresolved [47,48]. Four genera, containing about 210 species, are currently known: *Argulus* (Müller, 1785), *Dolops* (Audouin, 1837), *Chonopeltis* (Thiele, 1900) and *Dipteropeltis* (Calman, 1912). Only a few species are well-known: *Argulus foliaceus* (Linnaeus, 1758), *Argulus japonicus* (Thiele, 1900), and *Dolops ranarum* (Stuhlmann, 1891). *Argulus vittatus* was recently described in more detail following a scanning electron microscope-based study [49]. Most other branchiuran species remain poorly investigated [43,49].

**Figure 3:**
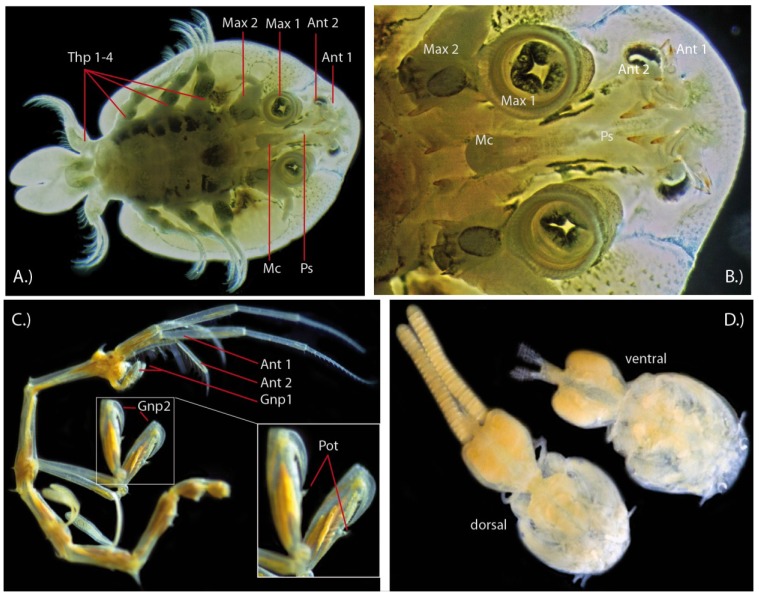
Other putative venomous crustaceans. (**A**) Branchiuran carp louse *Argulus foliacaetus* from ventral side; (**B**) *Argulus* mouthparts from ventral side. Thp 1–4 = thoracopods 1–4, Max 1 = maxilla 1 (maxillule) (with last segment modified to sucker disc), Max 2 = maxilla 2, Mc = mouth cone, Ps = Preoral spine (slightly disarranged by ethanol preservation); (**C**) Male skeleton shrimp *Caprella scaura* with pleiopods removed. Inset shows gnathopod 2 appendages, which bear the poison tooth. Gnp = gnathopod, Pot = poison tooth (**D**) Female sea lice or siphonostomatoid copepods. Collection reference numbers for specimens of the Natural History Museum London: *Caprella Scaura*, 1902, male, Inland sea, Japan (Amphipods, Caprellidea: NHMUK 1902.12.12.6/7). *Caligus rogercresseyi*, 2000, female, Puerto Montt Chile, Host *Eleginops maclovinus*, J. Carvaja: (Copepods, Siphonostomatoidae: 2000.1258-126). Specimens were photographed with a Nikon D200, Sigma 150 mm EX-APO Macro-lens and ring-flashgun units.

The first speculations about the use of putative venom were made rather early for the carp louse *Argulus foliaceus*, for which a structure located in the midline of the head and called a preoral spine or “Giftstachel” (“poison spine”) was described by Claus (1875). [50] Claus assumed an injection needle-like function for this spine, which originates from a different region of the head than the remipede maxillules that deliver their venom. In fish lice the maxillules are used to attach to the host, while the preoral spine derives from the head area between the second pair of antennae and the mouth cone, see also Figure 3A,B. The preoral spine is found in *Argulus* and *Dipteropeltis* [47,49,51,52] and supports a close relationship between these genera*.*

Different interpretations exist about the purpose of the preoral spine [51,52], as well as the related gland systems [51]. In general, *Argulus* and *Dipteropeltis* show two structures that are capable of injecting possible toxins into the host’s body. The first structure is the mouth at the bottom of a mouth tube (authors use the term proboscis and mouth cone interchangeably; we will refer to the term proboscis here). The mouth is composed of a labrum and labium with a pair of labial spines [47,49,52].

The second structure is the preoral spine, which comprises two different parts. The spine is approximately 750 μm long in an adult *Argulus japonicas* [52]. One proximal part is related to the mouth tube and is sheath-like, the distal part is stout, retractile and likely used to sting into the host tissue [47,49,51]. The spine contains a duct that opens subterminally on the dorsal side of the spine. A smaller ventrally located pore is probably the opening of a chemoreceptor [53].

Initial assumptions that the preoral spine is used to suck up body liquids and blood were disproven when it was shown that no connection exists to the oesophagus, see also [52,53]. It obviously functions by injecting secreted products into the host’s tissues through the large sub-terminal pore [53]. Saha and colleagues document a preoral spine related sac-like gland composed of four polygonal cells, which is in line with the classical view of Wilson (1902) [54] and Madsen (1964) [55] who described a *glandula paraeboscialis* that resembles the preoral spine gland described in Saha *et al.* (2011) [51]. However, Saha and coworkers state that the duct leading to the spine is ending blind at the glandular end, which is differently reported by Gresty and colleagues [53]. Both agree that a single duct connects the spine with the gland contrary to previous descriptions of a paired duct system [52].

It remains unclear how the glands associated with the proboscis are involved in secretion. Studies report a secretion via ducts into the proboscis and buccal lumen [51,52,53]. Swanepoel and colleagues describe that one duct is connected to the labial spines, which contradicts Madsen (1964) [55] that more glands are connected to the labial spines.

Striking is that branchiurans might have at least two systems to interfere with host physiology. First, a secretion into the buccal cavity by proboscial glands, second secretion via the preoral spine gland, and potentially via the labial spine associated glands.

New studies are clearly needed to illuminate the precise morphology of the branchiurans’ venom apparatus. Similarly the composition of the putative venom remains completely unknown. In two studies activity tests were conducted with extracts from dissected mouthparts of *Argulus* *coregoni* and *Argulus siamensis* that were injected into fish [51]. Interestingly the two studies contradict each other. Shimura and colleagues suggest a hemorrhagic response, but no hemolytic or cytotoxic effects, while Saha *et al.* report with similar experiments no hemorrhagic effects, but a clotting time delay after injecting the extract into fish. The preoral spine is additionally thought to inject an anaesthetic that might act as a vasodilator [51]. Recent publications cite both effects, lytic and vasodilatory effects [47,49]. Neither transcriptomic nor proteomic analyses exist that describe the expressed genes and proteins in the gland systems associated with the preoral spine or other mouthparts. No study attempted to investigate the effects of secreted products of these gland systems separately. This theme is similar to other neglected venomous taxa and will be discussed in more detail later in the section on robberflies. Learning more about the putative venom apparatus and venom of fish lice could be vital for practical applications used in marine and freshwater aqua farming, but also for shedding light on general aspects of venom and toxin evolution in crustaceans and euarthropods.

#### 2.2.2. Skeleton Shrimp (Caprellidae)

Caprellidae are also known as skeleton shrimps, and are also often referred to as ghost shrimps. The latter common name is misleading as “ghost shrimps” is also used for two other crustacean groups, the Thalassinidea and Palaemonidae, both decapod malacostracans. Caprellidae, however, belong to the large malacostracan order Amphipoda [56]. They are easy to identify by their typical slender elongated body, which is also denoted in the common name. Skeleton shrimps are benthic, cosmopolitan estuarine and marine crustaceans [46,57] that are found mostly in the littoral zone attached to different substrates ranging from macroalgae, tunicates, seagrass beds and artificial structures. Some of the caprellid species like the Japanese skeleton shrimp (*Caprella mutica*) or more broadly ranging *Caprella scaura* are invasive species, e.g., in the Mediterranean sea, which might be linked to their general association with artificial structures, including fish cages from aquaculture or fishery, and boat hulls [45,58]. The impacts of the emerging alien/invasive caprellid species are the subject of ongoing studies [44].

Caprellids show a gender-specific dimorphism. The males develop a pointed protrusion on their second gnathopods, which are appendages that are used as weapons in combat [58]. These structures on the second gnathopods are utilized in male to male competition and to inflict injury, see [59,60]. Interestingly, many pores were located on these protrusions or larger, pointed spines, and an extensive array of rosette glands and ducts leading to pores inside were documented [59]. Schulz and Alexander [59] discuss critically the claim by Wetzel in Lewbel [61] that this structure or “poison tooth” could anaesthetize prey using a toxin. The presence and nature of the putative venom has never been investigated, neither on the molecular, protein or chemical level, nor its delivery system on a more sophisticated morphological level. The possible presence of venom therefore remains unconfirmed [58,59]. Indeed, observations already exist that the mechanical damage by the tooth can impose lethal or harmful injuries, see [58]. The general structure of the “poison tooth”, which obviously plays an important role for competitive interactions clearly needs further investigation [60]. Interestingly, not all caprellid species have this “poison tooth”, it has been shown so far for the genera *Caprella*, *Paracaprella*, *Luconacia*, *Paradicaprella*, and *Aciconula* [59]. We might speculate that the success of invasive caprellids might be linked also to the use of putative venom. Invasive species like *Caprella scaura* and *Caprella mutica* possess a “poison tooth”.

If venom is indeed produced by males and used for male-male interactions this would represent a rare case of venom use in intraspecific competition. So far only platypus males and slow lorises [62] are known to apply venom in fights for mates between conspecifics.

#### 2.2.3. Copepoda

Species in the calanoid copepod family Heterorhabdidae are carnivores. Copepods in the genera *Heterorhabdus*, *Neorhabdus* and *Hemirhabdus* are thought to be able to produce and inject a toxic secretion into prey. *Heterorhabdus* species have the most specialized mandibular morphology to accomplish this. They possess a hollow, sharp-tipped mandibular tooth with a subterminal opening, which is associated with gland cells from the labrum. This configuration is reminiscent of a hypodermic needle, and Nishida and Ohtsuka [63] and Ohtsuka *et al.* [64] hypothesize that these small crustaceans use their mandibular teeth to inject toxic substances generated in the labral glands into prey to subdue it. A phylogenetic comparison suggests that these predatory habits have evolved from ancestors that were particle feeders.

Species in another group of copepods, the parasitic Siphonostomatoida (Figure 3D), are known to be able to produce and secrete pharmacologically active compounds that can affect the physiology of their fish hosts. The best studied species is the salmon louse *Lepeophtheirus salmonis*, which can have devastating effects on salmon farms. They settle on hosts by injecting a glue into the epidermis [65] and they feed on host mucus, epidermal cells and blood. They secrete or regurgitate a proteolytic cocktail that contains proteases (such as trypsin) [66] as well as substances that can modify the host’s immune system [66]. Ectoparasitic species therefore provide a potentially rich source of bioactive compounds. Under the new toxin terminology devised by Nelsen *et al.* [67] most ectoparasites can be classified as either venomous or toxungenous; they use a delivery mechanism to apply toxins to another organism either via a wound (venomous) or not (toxungenous). Hence the study of pharmacologically active parasite secretions can be considered a legitimate part of the science of venomics.

#### 2.2.4. Gnathiid Isopods

Although parasitism is a common lifestyle for isopods, almost nothing is known about the potential role played by bioactive molecules. An exception is a recent study on gnathiid isopods (*Paragnathia formica*), the juveniles of which are hematophagous ectoparasites of fish [68]. This study showed that crude extracts of juveniles have trypsin inhibitory and anticoagulant activities. The authors speculate that it is likely that the anticoagulants are expressed in the salivary glands of the isopods, but future studies to confirm this will be challenging because the gnathiid juveniles are just 1 mm long.

### 2.3. Neglected Venomous Insects

The insect order Diptera (true flies) is with Coleoptera (beetles), Lepidoptera (butterflies and moths) and Hymenoptera (ants, bees and wasps) one of the mega-diverse insect orders, and is one of the most species-rich and ecologically diverse group of arthropods. Diptera represents 10%–15% of all known animal species, including more than 150,000 described species [69,70]. Diptera includes important pest species that affect humanity in various ways as vectors for human and crop pathogens. Most of these are hematophagous (blood-feeding) and therefore by definition also venomous [4]. In particular, many non-brachycerans (formerly grouped in “Nematocera”, a clade that is at present considered to be paraphyletic, see also Lambkin *et al.* 2012 [69]), such as *Anopheles*, *Aedes*, *Culex* (Culicidae), and *Phlebotomus*, *Lutzomzya* (Psychodidae) have been extensively studied*,* as well as the brachyceran *Glossina* (Glossinidae). These studies included transcriptomic approaches to describe gene expression in their salivary glands [71], because they play an important role as pathogen vectors for diseases such as malaria, leishmaniasis, trypanosomiasis, sleeping sickness, and other protozoa and virus born infections (see also Table S1). However, the adults and larvae of some groups, especially brachycerans, are suspected of utilizing venom to overcome prey, but not much is known about their possible venoms.

#### 2.3.1. Robber Flies (Asilidae)

**Figure 4:**
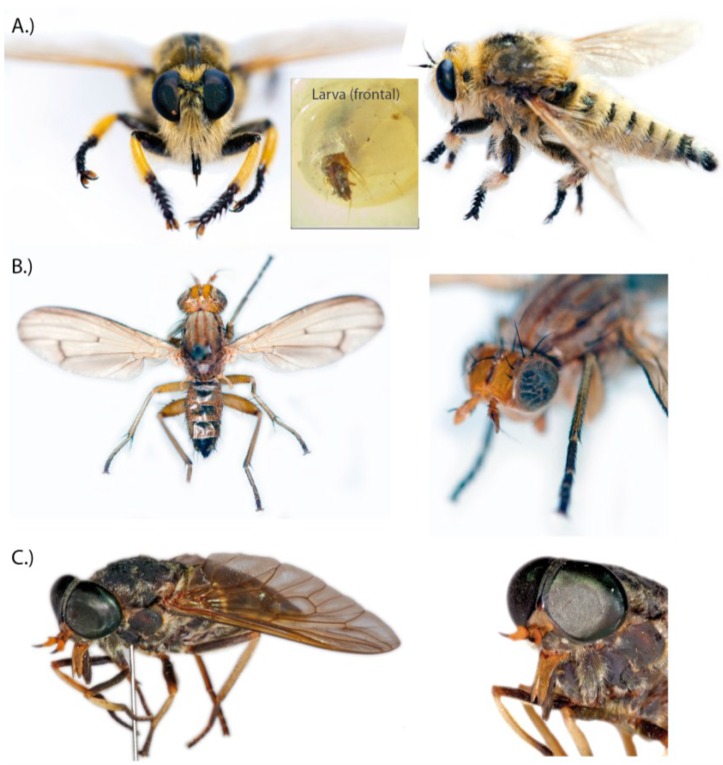
Examples of fly groups that have neglected, likely venomous species. (**A**) Robber flies (Asilidae): *Promachus leoninus* specimens and larva of a British robber fly; (**B**) Marsh flies (Sciomyzidae): *Tetanocera elata*; (**C**) Horse flies (Tabanidae): *Tabanus trigonus*, one of the species of which larvae pose a risk to rice workers in Japan. Collection reference numbers for specimens of the Natural History Museum London: *Promachus leoninus*, Turkey, Nurdagi Gecidi, 1960 (DIP3404, B35 193, Bactria, BMNH(E) 1237803). *Tetanocera elata*, United Kingdom, 1971, (DIP957, C57, 8, BMNH(E)1237801). *Tabanus trigonus* 1972, Japan, Kasumigaura Ibaraki (DIP3026B5, 189, Tabanidae, BMNH(E) 1237802).

Robber flies (Asilidae) represent one of the largest extant fly groups within Brachycera, comprising more than 500 genera with over 7000 species that can be traced back to the Albian age of the Cretaceous, ~112 million years ago [72,73]. Adult robber flies have a very robust, but mostly slender body, which is obviously an adaptation to their typical habit of preying on other insects either airborne or from a raised position. A synapomorphy of this group is the heavily sclerotized, tube like proboscis that envelopes a needle-like hypopharynx [74], forming a strong, lance-like stinging apparatus; see also Figure 4A. Another characteristic is that adults are strictly predators.

Observations tracing back at least to the mid-19th century [75] report that adult robber flies are capable of predating on larger and even venomous prey that seems to become paralyzed immediately when caught. Since then scientists have been interested to learn if robber flies possibly utilize venom to overcome their prey, which includes large-bodied insects from different orders, such as grasshoppers (e.g., [75,76,77]. Especially predation on economically important honeybee species (e.g., *Apis mellifera*) and other hymenopterans with venomous stings like wasps attracted some early attention [76,78,79]. These hymenopterans can defend themselves effectively by their stinger apparatus. Consequently one plausible assumption was that brute force alone is not sufficient for robber flies to catch prey, and that this might have to be aided by envenomation. The first attempts to test experimentally the presence of venom in asilids were conducted by Le Conte (1850), and in an extended approach by Whitfield [77] by comparing the time to death of grasshoppers that were stabbed with needles to those that were tackled by asilids. Both researchers concluded that the asilid’s bites kill prey dramatically faster, which implies that they deliver a substance that accelerates the death of the prey. Whitfield posed the question if saliva and toxin were identical or secreted by separate gland systems, a pair of thoracic glands and a pair of smaller labial glands that he described [77]. It is common for flies to exhibit two or more salivary gland systems associated with the mouthparts. Two pairs of glands were described early in the dipterans *Calliphora* and *Musca*, but also previously in 1900 for the asilid *Laphria*; see [80]. Whitfield proposed that the thoracic glands that terminate separately from the labial glands in the proboscis secrete a possible toxin [77]. Both structures were described later by Owsley, who studied further asilid species and concluded that a similar histological structure was present, but that the labial glands found in asilids could vary in their conformation [80]. For example *Promachus* shows much larger and longer labial glands then *Asilus* studied by Whitfield [80].

Kahan produced the first and to date the most thorough study of toxic effects and proteolytic activity of thoracic gland and stomach contents of asilid flies [81]. He compared the toxic activity of thoracic salivary gland samples in mice, locusts and protozoa of nine asilid species (*Promachus leoninus*, *Promachus griseiventris*, *Philonicus dorsiger*, *Machimus* sp., *Echtistus rufinervis*, *Neomochterus mundus*, *Stenopogon* sp., *Saropogon leucocephalus*, *Habropogon* sp.). Additionally the proteolytic activity of thoracic gland, labial gland and stomach content of *Promachus griseiventris* and only the stomach of *Philonicus dorsiger* were tested separately. Experiments in which gland tissue crushed in physiological salt solution was injected into locusts (*Locusta migratoria*) showed that the toxin in the thoracic glands of the species obviously varies in strength. The strongest effect was observed for *Promachus leoninus.* 1/128th fraction of its venom glands killed the locust. Strong effects were also observed for two other species, with the lethal dose of *Promachus griseiventris* and *Philonicus dorsiger* being 1/32th of the venom glands. All other asilids showed weaker toxin [81]. Salivary gland suspensions of *Machimus rusticus* and *Promachus dorsiger* were additionally tested for effects on vertebrates by intraperitoneal injection into mice. The putative venom of *P. dorsiger* acted more strongly, with the four glands approximately containing a lethal dose. Effects on the mice are: less activity, bristling hairs, labored breathing and body contractions, which hints at a neurotoxic component, and also matches the effects observed on locusts. Later studies by Musso and colleagues compared toxicity effects of further asilid species based on the methods of Kahan, but only thoracic gland material was used [81,82,83]. Musso and coworkers conclude similar toxic effects, but also varying strength between different taxa. Both studies [83] discuss also critically the difficulty of comparing the toxic effects on different species and of generalizing the experimental outcomes in terms of venom “units”.

Interestingly, Kahan obviously assumed, based on the work of Whitfield, that only the salivary thoracic glands secret possible venom, while the labial glands produce proteinaceous liquid. Proteolytic activity was tested separately for the labial glands, thoracic glands and stomach of *Promachus griseiventris.* The results showed that the labial gland had no proteolytic effect, contrary to the assumption by Whitfield [77]. Proteolytic effects were only observed for the liquids of the thoracic glands and the stomach [81]. Kahan concluded the thoracic salivary glands produce both toxic and proteolytic liquids, comparing them to the venom glands of snakes. These structures are of course not homologous. However, from morphological descriptions of snake venom glands it is well known that some are also composed of a main gland and an accessory gland. Based on morphological and histological data it was assumed for some time that the accessory gland might play an important role in modification and activation of the venom, as well as secreting specific toxin components [84,85]. The venomous effects of the labial glands of asilids remain to be investigated. Important toxins, including (non-proteinaceous) neurotoxins could be secreted by those glands. It could well be that the complexity of the asilid venom results from mixing the distinct secretions of the thoracic and labial glands, the components of which may interact and enhance the venom’s effects when injected into prey.

In contrast to the adult stages many fly larvae are predators, but detailed studies on dipteran larvae (including asilid larvae) that address the presence of potential venom are rare. A good overview of a few observations on the venomous effects of insects, including larval forms and dipterans, is given by Schmidt [86]. Interestingly, the adults of Asilidae are here described as the venomous stage of robber flies and their predatory larvae are not considered. However, two other important fly groups are addressed that show venomous larvae, the horse flies (Tabanidae) and the marsh flies (Sciomyzidae).

#### 2.3.2. Horse Flies (Tabanidae)

Tabanids are a large group of flies with over 140 genera and more than 4000 species, of which many are important live stock pests. They also affect humans as vectors that transmit various important disease agents such as the *Loa loa* worm, trypanosomes, *Bacillus anthracis* and many others [87]. The family Tabanidae represents one of the more ancient lineages within brachyceran flies and is subdivided into four subfamilies (Chrysophsinae, Pangoniinae, Scepsidinae and Tabaninae) [69,88,89]. Most of the economically important tabanids are found within Tabaninae and Chrysophsinae, with genera like *Tabanus*, often referred to as horse flies and *Chrysops*, also known as deer flies.

However, even more interesting is the story behind the larval venom from these flies. The larval venom is so far neglected, despite the fact that larvae of tabanids have since the 1930s been thought to utilize venom that reportedly paralyzes prey immediately. When bitten by tabanid larvae, nerves and muscles of larvae of *Galleria mellonella* (honeycombe moth) show no action currents and tissue was dissolved rapidly. These effects were interpreted as evidence for the presence of a neurotoxic and lytic venom [90,91]. Curiously, larvae of *Tabanus punctifer* have also been described to prey upon young spadefoot toads (*Scaphiopus multiplicatus*), by biting the toads with their rattlesnake like fang-shaped mouthparts from beneath, paralyzing the toads and dragging them partly into the substrate, in which the tabanid larvae wait for prey, to suck out the body liquids [92,93]. Laboratory experiments with larvae of *Tabanus punctifer* showed that they can overwhelm much larger and stronger prey such as the bombardier beetle (*Brachynus* ssp.) and crickets (*Teleogryllus oceanicus*) by partly paralyzing them [93]. The same effects were reported via a personal communication of R. S. Lane who describes that after a bite from a tabanid larva prey “cease all movement after one or two spasms” [86].

Another interesting aspect of this putative venom besides neurotoxic and lytic components seems to be a pain-inducing component. Several reports describe bites of some tabanid larvae as very painful, bee sting like [86]. It is known from Japanese rice workers that *Chrysops* and *Tabanus* larvae bites can cause pain for ten minutes to two days, accompanied by intense itching, erythema extending to 75 mm or more and lymph node swelling [94]. Interestingly, there are more recent reports of hobby entomologists’ discussions on specialized internet fora like [95] in which very similar symptoms are detailed and precisely described from people that were bitten handling tabanid larvae.

#### 2.3.3. Marsh Flies (Sciomyzidae)

Another group of flies that developed predaceous larvae that are assumed to utilize venom are the marsh flies (Sciomyzidae) [96]. It should be noted here that the common name can be misleading as in Australia some tabanid species are referred to as marsh flies too. Sciomyzids, however, feed on plant dew or nectar as adults and live in moist to wet habitats where courtship, mating and depositing of eggs takes place. Their larvae are predaceous on molluscs. For that reason this group is also called “snail-killing flies”. Sciomyzid life histories are well studied and a wide range of larval feeding habits are known, including parasitism, saprophagy and predation on terrestrial, semi-aquatic and aquatic snails, slugs, snail eggs, clams and some freshwater oligochaete worms [97,98,99,100]. The sciomyzid larvae have three stages of which the third instars often become more generalized predators, while in particular in parasitoid species, the first and second instars are very host specific [98,100]. Sciomyzidae is divided into three subfamilies, the Huttonininae, the Salticelloinae and the Sciomyzinae. New morphological data suggest that Sciomyzinae is monophyletic but subdivided into two tribes, the Sciomyzini and the Tetanocerini [98]. For a more detailed phylogenetic discussion see [100,101]. All larvae of Sciomyzini and Salticellinae are terrestrial, while some genera of Tetanocerini have also developed aquatic larval stages. Interestingly, Huttonini larvae remain unknown [98].

After obligate snail killing behavior of sciomyzids was first shown by Berg in the 1950s [102], it was later assumed based on observations that larvae of the last stage of some sciomyzids might use a neurotoxic venom component to paralyze and immobilize the much larger slug prey. Activity tests with isolated gland homogenates from the sciomyzids *Tetanocera plebeia* and *Tetanocera elata* showed that transmission of impulses from axons of the pedal nerves to the longitudinal muscles of the slug’s foot were blocked [86,97]. The proteolytic toxin that is assumed to be responsible for this effect was isolated from the salivary gland homogenates by Sephadex gel filtration [97]. It hydrolyzes casein implying that the isolated toxin might not represent the particular neurotoxin component. Neurotoxins normally block, activate or interact with voltage sensitive ion channels, nerve terminals and specific proteins or receptor proteins that are functionally associated to those structures, but neurotoxins normally show no proteolytic activity [4,103,104]. Slugs that received bites lasting less than 30 s become rapidly paralyzed, then recover in 15–20 min and subsequently die in 2–24 h, which indeed implies neurotoxic activity [96]. It was also reported that for *Tetanocera elata* the effect of paralyzing slugs is stronger compared to that of *Tetanocera plebeia*, though the position of the bite also seems to play a role [96]. Interestingly, a recent study has recorded the attacks of *Tetanocera elata* on the slug *Geomalacus maculusos* in laboratory experiments and documents the immobilization of the slugs 4–7 min after being attacked [105], though it needs to be mentioned that under natural conditions *T. elata* has so far not been reported to feed on this slug species.

An important aspect of the potential practical application of venoms is that soon after discovering the biology of sciomyzids they became promising biological agents that could be used against several snail borne diseases or snail pest species [97,100]. The high prey specificity of some marsh fly larval stages, in combination with their specific habitat requirements provide a solid base for fighting specific snail pests or disease vector species, in particular ones relevant to human health (e.g., for *Schistosoma*), but this area needs further investigation [100]. Several recent studies conclude that the sciomyzid fly species *Sepedon spinipes* (Scopoli) represents a promising biocontrol agent against fasciolasis [106]. Despite the fact that snail-borne diseases are relatively neglected diseases, fasciolasis has been better studied because it is an important livestock and human disease. This digenean trematode borne disease is caused by the common liver fluke (*Fasciola hepatica*) and *Fasciola gigantica*. Important intermediate host species are lymnaeid snails (*Galba truncata, Radix balthica*). Sciomyzids showed a huge potential to break the transmission cycle by eliminating the snail hosts [106,107]

#### 2.3.4. Further Neglected Fly Groups

Cecidomyiidae (aka Cecidomyidae) are commonly known as gall midges or gall gnats, and is a family of flies that are mostly known for several pest species like the Hessian fly *Mayetiola destructor*. Large numbers of gall midges, however, have larvae that are predaceous and natural enemies of other crop pest species, including aphids, spider mites, and hemipterans (“whitefly”). An economically important species that is used as a biocontrol agent especially for “biologically” farmed greenhouse crops is *Aphidoletes aphidimyza.* Several garden and agricultural distributors list products that contain its larvae, which can indeed significantly reduce aphid infestations and apply these as alternative biocontrol agents.

Only one older publication written in German describes a venomous bite of these larvae when they attack preferably the leg joints of aphids [108], paralyzing the aphid and then feeding on their body liquids. The prey stays immobilized even when larvae release the aphids immediately after the bite. Morphologically no oesophageal or buccal glands have been described but only well-developed larval salivary glands [108]. In Mayr’s study fifty glands and one complete digestive tract were homogenized in 5 μL buffer, then centrifuged at 17.000 rpm for 3 min. Five to ten aphids (*Myzus persicae*) were injected with 4–6 nL of the two homogenates and in parallel a control group only received buffer liquid [108]. The salivary gland liquid resulted in paralysis of all aphids after 2–10 min. While the digestive tract homogenate showed effects only after several hours. The control group did not experience higher mortality. The salivary gland solution had the same effects in basic or acidic conditions (pH 6.8, 5.5, 4.8). Interestingly, its effectiveness also applies to heteropterans (*Anthoceris nemorum*) and dipteran adults (*Drosophila melanogaster*). Observations showed that the average time until a complete and irreversible paralysis takes 1–2 min in aphids. If this paralysis happens after more than 30 min its effect is in most cases reversible. Paralysis of extremities occurs without any observable signs of hyperactivity. *In vitro* tests of proteolytic activity were negative for the salivary gland solution, but positive for the digestive tract liquid. An assumption by Mayr was that phenoloxidase, which is traceable and inhibited by a phenylthiocarbamide reaction, might function as a toxin that plays a role in immobilizing the prey. Alongside its role in the sclerotisation and melanisation of the cuticle to increase its durability, phenoloxidases are known to play a role in the defense of arthropods against microorganisms. The detection of phenoloxidase and its hypothesized toxic function might remind readers of the erroneous hypothesis, discussed earlier in this paper, that remipedes might have a venom with phenoloxidase activity. Injecting aphid phenoloxidase derived from fungi, however, needed a ten times higher concentration to show effects that were less stronger and induced paralysis only after hours. The described effects indeed suggest that a highly potent neurotoxin acts as one major component within the gall midge larvae venom, which also seems to affect other insect species.

Vermileonidae is a small group of flies that is still being discussed regarding its origin within flies. Older studies related them to the Rhagionidae (snipe flies). Vermileonid species are hard to find, which may be related to their unusual biology. The larvae of this group are also known as wormlions, which prey on other insects by trapping them in cone-shaped pits in sandy areas. This mode of predation is remarkable and is convergent to that of neuropteran antlion larvae. Only one reference that larvae of this fly group might be venomous is cited in Schmidt [86], to the effect that the salivary glands of the larva of a species of vermileonid resemble those of the adults of asilids. The morphological adaptations, which are shown in both sandlions and wormlions include of course structures and hooks that can prevent the escape of prey without the need for envenomation. However, venom that ensures an immediate paralysis of prey may be useful in areas with a lower density of prey and in the remote and extreme habitats of this group (thermophilic, sandy areas). Yet, the general biology of wormlion larvae has still not been investigated in detail.

### 2.4. Neglected Centipedes

**Figure 5:**
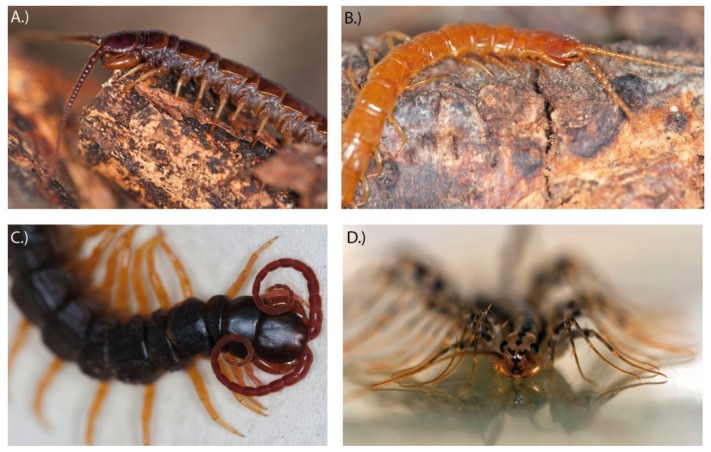
Representative species of neglected but common centipede groups. (**A**) *Lithobius forficatus* (Lithobiomorpha); (**B**) *Cryptops* sp. (Scolopendromorpha); (**C**) *Alipes grandidieri* (Scolopendromorpha) (**D**) *Scutigera coleoptrata* (Scutigeromorpha).

Centipedes are a group of over 3000 species of venomous invertebrates (Figure 5). They were largely neglected in venom research until very recently. However, a series of papers published in the last few years has begun to change this situation, revealing fascinating new details about their predatory behavior and morphology, venom composition, and the pharmacological effects of centipede venom toxins [8,10,109,110]. Here we highlight several of these recent advances in order to stimulate further new research.

Even though centipedes represent one and a half as many venomous species as do scorpions [111] the venom literature on scorpions is at least an order of magnitude larger (based on a Web of Science search in August 2014). No doubt this is chiefly due to the lesser medical relevance of centipedes. Although centipede bites are common in certain parts of the world, and can be very painful, they only very rarely pose any serious health risk. Just three well-documented fatalities have been ascribed to centipede bites [112]. In contrast, scorpions are a major public health problem in the tropics and subtropics and kill thousands of people every year [113].

The basic morphology of the venom delivery apparatus of centipedes is fairly well understood [109,112,114,115]. Venom is synthesized and stored in a pair of venom glands that are located inside a pair of sharp-tipped and generally robust forcipules (maxillipedes), but in some centipede species the venom glands extend posteriorly into the body. Venom is delivered from the glands via a venom duct that opens subterminally on the forcipules. The fossil record suggests that forcipules have been a key feature of the centipede body plan for at least 400 million years [115,116].

Several recent studies have shed new light on the origin and evolution of the venom apparatus [8,10,109,115,117,118]. These studies suggest that the venom glands originated as patches of glandular epidermal epithelium and its adjoining cuticle that became increasingly invaginated into the interior of the forcipules. During centipede evolution the forcipules have changed their overall shape and modes of articulation, becoming increasingly less like walking legs from an ancestral condition reminiscent of extant scutigeromorphs (house centipedes). Morphological changes involved the first article of the distal part of the forcipules (trochanteroprefemur) becoming more stout and robust, the extreme shortening of the middle articles of the forcipules, fusion of the forcipular coxae (present in scutigeromorphs) into a broad coxosternite (present in the other orders), a shift in the articulation of the trochanteroprefemur with the proximal coxosternites to a more anterior position, restricting the movement of the forcipules to the horizontal plane. Dugon *et al.* (2012) [118] hypothesize that these morphological changes broadly correspond to an ecological shift from living and hunting in more open spaces (like extant scutigeromorphs) to more enclosed spaces, such as leaf litter, rotting wood and soil.

Centipedes do not inject venom into prey indiscriminately. Dugon and Arthur [119] showed that *Scolopendra subspinipes mutilans* adjusts its venom delivery to the type of prey and the amount of venom available in the venom glands. Animals with depleted venom glands are less likely to attack available prey, or when they do, they are more likely to release the prey than when their venom glands are full. Moreover, it takes a longer time for centipedes with recovering venom glands to be willing to attack larger prey than smaller prey. Finally, Dugon and Arthur [119] observed that after first contact the centipedes manipulate their prey so as to preferentially envenomate the head and thorax of insects, rather than their abdomen. This would ensure that venom is delivered as close as possible to target areas (cephalic and thoracic ganglia) responsible for controlling locomotion.

Studies investigating the composition and bioactivities of centipede venom until very recently were focused on a few species in the scolopendromorph genus *Scolopendra*. Rates *et al.* (2007) [120] was the first study that attempted to go beyond the characterization of single centipede venom toxins. They reported that the crude venoms of two species of *Scolopendra* contained proteins of more than 60 distinct molecular masses, and they determined the *N*-terminal amino acid sequences of 10 venom peptide families. Around the same time the study of Malta *et al.* [121] was the first to expand the analysis of the bioactivity of centipede venoms to species in two other scolopendromorph genera, *Otostigmus* and *Cryptops*. Finally Gonzalez *et al.* [122] sequenced the first full-length transcript of a centipede venom toxin. The publication of five papers since 2012 has dramatically boosted our understanding of the composition and bioactivities of centipede venoms by using a combination of high-throughput transcriptomic and proteomic methods [8,10,123,124,125] focused on two different subspecies of *Scolopendra subspinipes* and *Scolopendra viridis*, and revealed a great diversity of neurotoxins, as well as a smaller diversity of enzymes and venom allergens. Interestingly, Yang *et al.* [110] identified a peptide in the venom of *S. subspinipes mutilans* that selectively inhibits voltage-gated sodium channel Na_V_1.7 so that it acts as a powerful analgesic in mice. Earlier in 2014 Undheim *et al.* [8] published a benchmark paper that reports the first application of transcriptomic and proteomic techniques to characterize the venoms of six centipede species, five of which are scolopendromorphs in three genera (*Scolopendra*, *Cormocephalus*, and *Ethmostigmus*) and one scutigeromorph (*Thereuopoda* sp.). Their study revealed the diversification of a highly complex cocktail of enzymatic and non-enzymatic venom peptides and proteins, including peptidases belonging to different families, chitinase, hyaluronidase, phospholipase A_2_, and a great diversity of cysteine-rich peptides, many of which may turn out to function as neurotoxins. Interestingly, the scolopendromorph toxin cocktails were much more complex than that of the scutigeromorph, especially with respect to the toxin peptides, with the scutigeromorph expressing only five peptide families (three of which uniquely), and the scolopendromorphs expressing 28 peptide families. Moreover, another paper of Undheim and colleagues [10] showed that a number of the scolopendromorph venom toxins are encoded by multidomain transcripts that are translated into more than one peptide. This is in striking contrast to transcripts coding for a single mature toxin, which is the norm for arthropod toxins.

The application of -omics techniques has now placed centipedes firmly on the map of comparative venomics. The recent sequencing of the genome of the geophilomorph *Strigamia maritima* provides another valuable resource for centipede venomics. However, the diversity of sampled species remains very small, and only covers representatives of two of the five extant orders.

### 2.5. Arachnida

Arachnid venoms have evolved at least three times in the orders Aranaea (spiders), Scorpiones (scorpions), and Pseudoscorpiones, and possibly a fourth time in the form of hematophagous secretions produced by ticks [126] (order Acari). Although the composition and biology of spider and scorpion venoms are among the most studied and best understood of all animal venoms, [127,128] pseudoscorpions have been almost completely ignored in venomics studies. This is especially notable because pseudoscorpions represent a greater diversity of venomous species than do the scorpions.

#### 2.5.1. Pseudoscorpions

**Figure 6:**
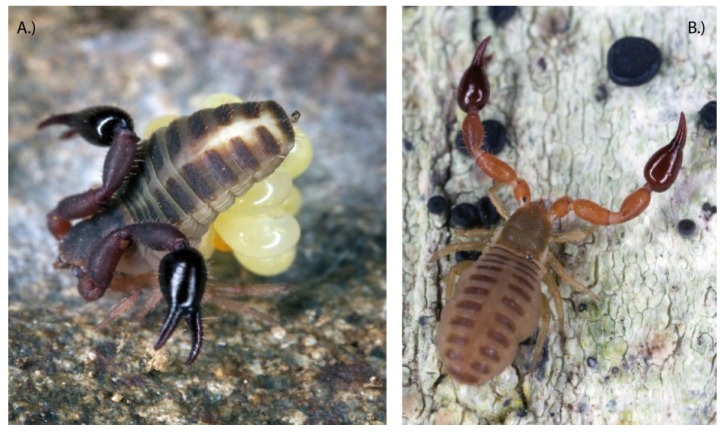
Pseudoscorpions. (**A**) Unidentified pseudoscorpion from New Zealand guarding its eggs; (**B**) Unidentified pseudoscorpion from New Zealand. Copyright for both photos is with Gonzalo Giribet, and are reproduced with his permission.

**Figure 7:**
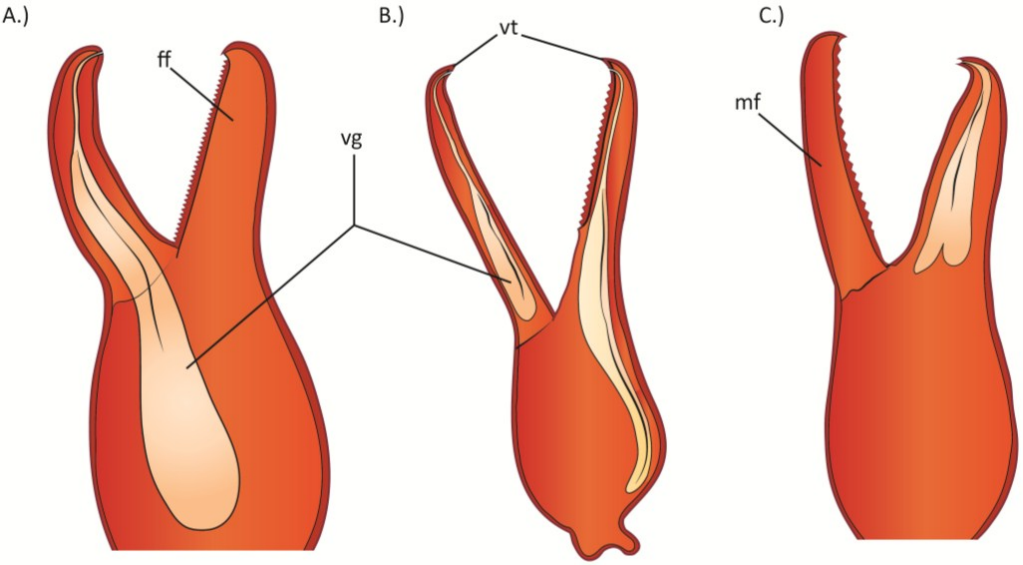
The venom glands in the palpal hand of: (**A**) *Cordylochernes macrochelatus*; (**B**) *Shravana laminata*; (**C**) *Neobisium flexifemoratum*. ff = fixed finger; mf = moveable finger; vg = venom gland; vt = venom tooth. Figure redrawn and modified from Weygoldt [130].

The arachnid order Pseudoscorpiones is represented by more than 3500 described species of generally small bodied (0.5–5 mm adult body length) animals [129]. Pseudoscorpions (also known as chelifers) inhabit many terrestrial habitats, particularly leaf litter, soil and under tree bark [130,131] Some species, such as *Chelifer cancroides* (popularly referred to as the book scorpion), can be found in houses worldwide, as well as in beehives throughout Europe [130,132,133]. Pseudoscorpions generally have squat bodies divided into an anterior prosoma, which lacks outward signs of segmentation, and an overtly segmented opisthosoma (Figure 6). The prosoma carries six pairs of appendages. The first pair are called chelicerae, and they form two-segmented chelae. The chelicerae are followed by a pair of chelate pedipalps, and four pairs of walking legs. In the vast majority of pseudoscorpions (about 2800 of the 3500 species) the chelae of the pedipalps house venom glands that open on either the fixed or movable fingers of the chelae, or both (Figure 7). Molecular phylogenetic analysis suggests that venom glands have evolved once within the pseudoscorpions at the base of a clade Iocheirata (“poison hands” according to its Greek etymology) [131,134]. The phylogenetic distribution of pedipalp morphology suggests that venom glands opening on both pedipalp fingers is the primitive condition, with several lineages losing the venom glands from either finger independently. However, no iocheiratan species has completely lost its venom glands, which suggests that they are an important pseudoscorpion adaptation.

Pseudoscorpions are thought to be generalist arthropod predators, but evidence suggests that pseudoscorpions can have a preference for certain prey species. Turk [135] noted that *Sphenochernes schulzi* kills and feeds upon leaf cutter ants in the genus *Atta*, but while it also killed ants in other genera (*Formica* and *Camponotus*) the pseudoscorpions refused to feed on these species. Interestingly, such prey specificity is promising for the potential use of pseudoscorpions in pest control. Fagan *et al.* and Read *et al.* [132,136] discovered that two native pseudoscorpion species from New Zealand, *Nesochernes gracilis* and *Heterochernes novaezealandiae* readily feed on aphid and fruit fly larvae, as well as *Varroa* mites, which are a worldwide parasite of honeybees. However, these pseudoscorpions do not attack bee larvae, pupae, or adults, so that a small number of pseudoscorpions can control the number of parasitic mites in a beehive.

Their venom may help pseudoscorpions subdue relatively large prey despite being small, especially in species that engage in cooperative hunting. Communal hunting allows pseudoscorpions from different families, such as *Paratemnoides enlongatus* and *Sphenochernes schulzi* to overwhelm and consume diverse prey, including beetles, millipedes, termites, and heavily sclerotized ants [135,137,138]. Available evidence suggests that the injection of venom plays an important role in predation. Rapid paralysis (within seconds) and death (within minutes) of flies or ants captured by pseudoscorpions has been reported [130,137], as well as slower death preceded by convulsive twitches of the prey [135]. We are aware of only a single experimental study into the effects of pseudoscorpion venom. This study showed that the crude venom of *Paratemnoides elongatus* affects the uptake and binding of neurotransmitter in rat brain preparations [139]. However, nothing at all is known about the composition of pseudoscorpion venom.

Given our fundamental ignorance with respect to most aspects of their venom-related biology, pseudoscorpions are an especially promising target for studies in comparative venomics. Moreover, the large number of pseudoscorpion species with venom glands represent a potential reservoir of novel bioactive compounds that surpasses that of scorpions.

#### 2.5.2. Camel Spiders or Wind Scorpions (Solifugae)

Solifugae are often still reported to possess a strong unknown venom despite the fact that species of this group do not possess venom glands like spiders. However, only one scientific publication exists that reports venomous effects of *Rhagodes nigrocinctus*, which is an endemic solifuge species in the Chingleput district, Tamil Nadu, India. On two pages the authors describe sets of epidermal glands along the tip of the chelicerae, which they assume produce venom [140]. One stained histological section through the anterior region of a chelicerae and a summarizing drawing of that area is shown. Experimentally, the glandular regions of the chelicerae tips were cut, ground with distilled water and centrifuged. The resulting supernatant was separated and 0.1 mL injected hypodermically into ten *Hemidactylus* geckos. The same experiment was conducted for non-glandular regions of the chelicerae. Seven of ten geckos became paralyzed and recovered after 48 to 72 h when injected with the liquid. The authors also conducted some biochemical tests with the glandular liquid indicating the presence of 5-hydroxytryptamine (5-HT). They propose that the pain caused by solifuges’ bites might be related to the presence of 5-HT, which is also found in the venom of scorpions.

The general idea that epidermal glands might produce toxins that are used as venom is interesting from an evolutionary perspective. Secretion of toxins via epidermal glands is very common as a defense strategy, for instance in amphibians which have some of the strongest toxins, such as that of the golden poison dart frog (*Phyllobates terribilis*). To modify this gland system to a venom delivery system as proposed by Aruchami and Rajulu for *Rhagodes* would be an interesting evolutionary scenario.

However, this topic clearly needs a more thorough biochemical and proteomic investigation. An interesting aspect of this study is that the glands apparently open via setae. A somewhat similar situation was described for glandular setae located on the maxillules and maxillae of remipedes [141], which the authors speculated may have a role in feeding by secreting toxic substances that could kill sediment dwelling little organisms that the remipede could then filter out.

Solifuges rely on their strong bites, which are similar in strength to scorpions [142]. However, it is doubtful that their setae are strong enough to inject venom into prey. Perhaps such glands and their secretions have a different, perhaps antibiotic-antimicrobic, function, similar to skin secretions of amphibians. In any case these open questions about the epidermal glands and possible secretion in Solifugae is interesting for further investigations using state of the art -omics and computer tomography scan based methods.

## 3. Lophotrochozoa (=Spiralia)

The large bilaterian clade Lophotrochozoa houses about 14 of the traditional metazoan phyla. It arguably represents the greatest amount of body plan disparity found in the animal kingdom. It includes microscopic and macroscopic sessile forms, such as entoprocts and brachiopods, motile marine and fresh water meiofauna such as gnathostomulids and micrognathozoans, as well as the four well-known motile and mostly macroscopic groups Mollusca, Annelida, Nemertea, and Platyhelminthes. Remarkably, specialized predators that use bioactive compounds to capture macroscopic prey have evolved independently in all four of these groups. However, morphologically conspicuous venom glands that produce complex proteinaceous toxin cocktails are only found in several groups of cephalopod and gastropod molluscs, and in several species of polychaete annelids. In contrast, nemertean and platyhelminth toxins used for predation are principally non-proteinaceous compounds such as the alkaloid tetrodotoxin.

### 3.1. Annelida

Annelids are closely related to Mollusca, Nemertea, Bryozoa, Brachiopoda and Sipuncula, which have all undergone an expansive radiation since the Cambrian [22,143,144]. Annelids are now considered one of the main groups of interest in the area of “bioprospecting”—the identification and analysis of biomolecules synthesized and secreted by various organisms spanning all domains of life [111]. The phylum Annelida comprises ~21,000 described species which are characterized by diverse and disparate morphologies. They can be found in a wide variety of habitats, including marine, freshwater and terrestrial environments. Despite the wide range of body plans within the group they are united by a number of shared morphological features, most notably chaeta (setae) and external cuticle and internal organ segmentation [145]. To date, phylogenetic relationships within Annelida and closely related groups await robust support [146].

Annelids display a wide range of lifestyles, particularly in relation to their mode of feeding. Feeding behavior is tightly correlated with the morphology of particular groups, and hence so too is the occurrence of venom use [147]. For instance, many marine polychaete groups exhibit predatory feeding facilitated by large jaws (e.g., *Glycera*), sometimes with associated venom glands, while other terrestrial groups are partially restricted to a parasitic lifestyle as exemplified by the well-known leech (e.g., Hirudinea). However, the vast majority of polychaete or oligocheate annelids are filter or surface deposit feeders of fine particulate matter (e.g., *Sabella*, *Terebella*). Confirmed venomous annelids with functionally active venom and a well-adapted venom delivery apparatus represent compelling curiosities of convergently evolved venoms in Annelida [3].

Annelids have a range of human applications and wide economic and ecological significance. For instance Charles Darwin highlighted the importance of earthworms in the recycling of nutrients and aeration of soil substrates [148]. Several groups within the annelids are utilized for their economic and medicinal benefits. Most notable is the medical practice of leech application (e.g., for the removal of a hematoma or “stagnant blood”) to patients with a range of ailments due to leech anticoagulation factors [149,150]. In terms of economic value, annelid worms represent a staple of the fish bait industry. Along the eastern coast of North America, for instance, there exists a prominent multimillion-dollar bait industry, in which large quantities of glycerid bloodworms (*Glycera dibranchiata*) are harvested [151].

Venom use in annelids is poorly studied. The best known venomous annelid group is that of the parasitic leech (Hirudinea). One of the most prominent hematophagous animal groups, leeches express a number of powerful anticoagulant proteins and peptides in their oral secretions that aid their feeding and digestion of a vertebrate blood meal [152]. Yet, although some venom researchers may disagree on whether or not hematophagous oral secretions such as those produced by leeches and blood sucking ticks, constitute true “venom” we consider these oral secretions as true venoms in line with recent definitions of toxic biological secretions [67].

An obscure annelid group capable of delivering active toxins to target organisms is that of the Amphinomidae, which are also known as “fireworms”, so named after the dense mat of skin-irritating chaetae covering their dorsal surface, or concentrated locally on the parapodia. Similar to these “non-biting” toxic polychaetes are others belonging to the genus *Aphrodita*, also known as “sea mice”, which share the same feature of defensive bristles along their dorsal surfaces, but only in sea mice these bristles are accompanied by far thicker spines capable of inducing considerable pain [153].

Jaw-associated glands are found in polychaetes in the family Glyceridae (bloodworms in the genera *Glycera*, *Hemipodia* and *Glycerella*) and in several families of scale-worms (families: Acoetidae, Pholoidae, Polynoidae, Sigalionidae, Pisionidae) [151,154]*.* Bloodworms can be considered truly venomous as they possess a complex venom delivery apparatus capable of injecting potent biologically active venom cocktails. The current state of our understanding of polychaete venom differs between bloodworms and scale-worms. While bloodworms have received thorough investigation of bioactivity and recently the first transcriptomic profiling [155], knowledge of scale-worm venom is non-existent. Despite these groups both possessing a venom delivery apparatus, there exists a disparity in overall morphology between scale-worms and bloodworms, with scale-worms occupying a much larger morphospace and body size between families compared to the more homogeneous bloodworms.

#### 3.1.1. Leeches (Hirudinea, Clitellata)

Hirudinea, more commonly known as leeches, is a group of annelids belonging to the sub-phylum Clitellata. Fine resolution for the phylogenetic placement of leeches (Hirudinea) remains contested [156], but it is generally accepted that they are closely allied within a likely paraphyletic Oligochaeta [146,157]. Although technically a member of Oligochaeta (meaning “few cheatae”), chaetae are absent in Hirudinea. In addition Hirudinea also display a more compact segmentation, muscular body with fewer segments, greatly reduced body cavity and single sucker at either end [149,158]. Leeches predominantly exhibit a predatory or parasitic lifestyle. The majority of Hirudinea species are hematophagous (bloodsucking), usually parasitizing a range of hosts including invertebrates and vertebrates. The most noteworthy hosts are warm-blooded mammals as they produce high volumes of nutrient rich material to support minimum body size [159,160].

Mainly occurring in freshwater environments, where they are common on the bottom or on low hanging foliage near slow flowing rivers, ponds, and swamps, leeches can also be found in marine and terrestrial environments [149,161]. The earliest documented leech species was that of the well-known central European medicinal leech *Hirudo medicinalis* (Euhirudinea) first described by Carl Linne in 1978. Traditionally, the phylogenetic history of Hirudinea has been approached almost exclusively through analyses of morphological data, until more recently larger molecular data sets have been applied to the group challenging the traditional monophyletic status of Hirudinea (See [162,163]). The earliest accepted Hirudinea representative dates back to only the mid Mesozoic based on purported reproductive vesicles shown to be most similar to that of living Hirudinea cocoon egg cases [146,164].

Of the neglected invertebrates described throughout this review, leeches will conjure a vivid image in peoples’ minds likely attributable to their historical depiction as voracious blood feeders. Unsurprisingly leeches have long been utilized for medicinal purposes in western culture, dating back as far as early centuries AD [150,163,165]. Leech have been purported to alleviate a wide range of human ailments e.g., fever, insomnia, ulcers [166]. Today one of the most common forms of medicinal leech application is to relieve post-operative venous congestion in patients recovering from tissue flap and replantation surgery [150,163]. Popularity of medicinal leech application stems directly from their intense feeding behavior and properties of their saliva that is secreted into a wound, specifically potent bioactive anticoagulatory proteins and peptides, in addition to anti-inflammatory and pain suppression components [160]. Not only do these proteins and peptides prevent blood clotting during phlebotomy (blood-feeding), but they also maintain a blood-meal in a suitable liquid state during the long period of digestion required post feeding [167].

Feeding behavior (e.g., parasitic, hematophagous *vs.* non-hematophagous, predatory) and by proxy the bioactive components comprising the oral secretions of leeches likely differ depending on the morphology of the feeding apparatus. On this basis, Hirudinea can be generally dived into three main orders: Acanthobdellida (oral sucker and jaws absent), Rhynchobdellida (jawless, bearing a strong muscular proboscis)*,* and Arhynchobdellida (mostly jawed leeches, but lacking a proboscis). *Hirudo medicinalis* is a prime example of a leech well adapted for hematophagy*.* Housed inside the anterior oral sucker are three independent jaws each comprised of a row of calcified teeth, which are rhythmically moved in opposite directions to create a “saw-like” action, resulting in destruction of superficial blood vessels and the acquisition of free flowing blood which is then sucked into the crop [168]. Leeches need to avoid being detected to maintain feeding. This is achieved via the secretion of anti-stimulatory and anti-inflammatory proteins and peptides [160]. These secretions flow from unicellular gland cells connected to the base of the jaws via elongated channels originating from the gland cells [169].

Of the three Hirudinea orders, Acanthobdellida appear to be the most peculiar, in that they have only two known living representatives *Acanthobdella peledina* and *Acanthobdella* *livanowi.* Possession of characters like setae have been cited as evidence for a status of “living relic” between extant oligocheates and leeches [170], which also is supported by molecular data [171]. Acanthobdellid leeches are known to be semi-permanent ectoparasites restricted almost exclusively to salmonoid fish [149,170,172]. Despite belonging within Hirudinea, to date acanthobdellid leeches have not been confirmed to be hematophagous. Conversely, evidence such as small feeding marks left on fish hosts, to the absence of fluid lacking resemblance to blood in their oesophageal tract, and insufficient tissue penetration support an epidermal feeding hypothesis [172]. For these reasons, it seems unlikely that acanthobdellid leech possess functionally active oral secretions comparable to that of true leeches (Euhirudinea), Rhynchobdellida and Arhynchobdellida [149,158]. However, no molecular data derived from acanthobdellid oral secretions have been obtained, and so this question remains currently unanswered.

Initial investigations into the secreted antiplatelet proteins produced by non-blood-feeding (Rhynchobdellida*:* glossiphoniid) leeches, have shown that although glossiphoniid leech are not true blood-feeders, they do possess and express eight homologs of a known leech antihaemostatic protein “LAPP” [167]. This finding stemmed from genomic annotations and remains unverified transcriptomically, however it is likely to be verified, as these homologs possess the necessary signal-peptides required for secretion [173].

To date the vast majority of molecular data available that characterize components in the oral secretions of leeches are derived from a subset of Euhirudinea. Subclass Euhirudinea consists of nine principal families that are traditionally divided into two orders mentioned previously (Rhynchobdellida and Arhynchobdellida) *sensu* Sawyer (1986) and Apakupakul (1999) [161,174] (but see also [156,163] for revised classifications of Hirudinea families). Currently there are two important analytical barriers impeding the thorough investigation of leech oral secretions (or salivary venom toxins): the inadequate taxon sampling across all major Hirudinea families, and the sequencing technology employed to characterize expressed venom toxins. Data derived within these two orders is largely biased towards the terrestrial blood feeding leeches, where the medically utilized type species *Hirudo medicinalis* resides. Specifically jawed leech (Hirudinea: Gnathobdellida), including *Macrobdella decora* (North American leech), *Hirudo verbena* (European medicinal leech) and *Asiaticobdella fenestrate* (African medicinal leech) are currently the only species that have received in depth molecular investigation for putative toxins [150,165]. In addition, data for these species was derived using older 454 Titanium pyrosequencing technology, providing shallower sequencing depth compared to that of more recent Illumina based sequencing by synthesis NGS (Next Generation Sequencing) technology. Furthermore, EST analyses of salivary tissue masses taken from these three leech species have only covered three out of the five families (~800 species) within the Suborder Hirudiniformes [150,162].

Medical application of leech has been and still is commonly practiced. Leeches require a suite of anticoagulant proteins to aid in feeding of a bloodmeal. Leech anticoagulants play a vital role in the interference of normal thrombus (blood clot) formation during various stages of the coagulation cascade, thereby increasing their ability to feed for extended periods of time [175]. Transcriptomic analyses of three medicinal leeches in the recent publications of Min *et al.* [165] (*Macrobdella decora*) and Kvist *et al.* [150] (*Hirudo verbena*, *Asiaticobdella fenestrate*) revealed a host of toxins families related to anticoagulant activity. Toxin families identified in these taxa include salivary proteins that are known to function in a variety of antagonistic pathways related to Factor Xa inhibitors, thrombin inhibitors, elastase inhibitors, plasmin inhibitors, and kazal-type serine protease inhibitors [166]. A list of commonly identified leech anticoagulant proteins and the associated studies that originally identified them can be found in [166].

Prior to these transcriptome-based analyses, studies that previously identified anticoagulant proteins in leeches (e.g., Hirudin, Bdellin, Destabillase, Cystatin, LAPP, Hirustatin, Saratin and Manillase) were restricted to sequencing of single or limited sets of toxin transcripts. Differently from those early studies Min *et al.* [165] and Kvist *et al.* [150] first used partial and whole EST libraries to screen for putative leech salivary toxins. These analyses showed that although there are differences in the repertoire of salivary proteins identified in each species of medicinal leech, the majority of known leech anticoagulant proteins were common to all three taxa. In addition, evolutionary analyses conducted on leech salivary gland transcript sequences by [150] provided some well needed phylogenetic framework to elucidate the relationships of some of the most commonly evolved leech anticoagulant proteins. Despite these efforts, in general there was little concordance between analyses of evolutionary histories of leech anticoagulants to previous hypotheses of leech phylogeny [163,165,176].

#### 3.1.2. Bristleworms (Amphinomida)

Amphinomida is a clade of marine polychaetes, referred to as “bristle worms” or more commonly as “fireworms”. The common names for members of this group derive from the presence of brittle chaetae that upon contact with skin break off and inject an inflammatory-causing substance known to cause a painful burning sensation [177]. To date, there are approximately 200 described species, and 25 genera thought to comprise Amphinomida [178]. Amphinomids have a circum-global distribution, and normally frequent warm littoral waters, such as shallow tropical seas, but also occur down to abyssal depths [178,179,180]. In terms of their ecology, this group of polychaetes is known to be tightly associated with coral reefs, in which they exist mainly as scavengers or slow predators on sessile prey such as sponges, cnidarians and ascidians [147,181]. A ventral pharynx, which is highly muscularized and forms a ventral proboscis and facilitates feeding in Amphinomida, which in predatory species can act as a rasping apparatus [178].

Amphinomida are easily recognizable from their characteristic chaetae. These defensive chaetae come in a wide variety of shapes and sizes (See [178]), including bifurcate, harpoon, spines and capillaries which are usually arranged in a row or in clusters and are bristled up when the animal is disturbed [153]. The majority of chaetae are hollow and highly calcified fragile structures, and can become imbedded in a wound upon light contact. Chaetae present on amphinomids have long been suspected to be responsible for the delivery of an active poison (See Eckert [182]), or potential neurotoxin [183]. The literature on the active toxin(s) produced in Amphinomida is scant. Existence of glandular cells believed to produce and supply an active neurotoxin to the chaetae was hypothesized, however this failed to be verified upon analysis by electron microscopy [182]. Despite lack of verification of a producing tissue, existence of an active toxin has been independently confirmed [184].

Recently an active toxin in amphinomid chaetae has been identified and named “complanine” after the species *Eurythoe complanata* sampled for identification of the unknown toxin. Complanine is a non-peptidic carbon-based compound, and was shown to activate protein kinase C (PKC) in the presence of Ca^2+^ and TPA (12-*O-*tetradecanoylphorbol 13-acetate) [177]. This activation leads to a signal transduction cascade resulting in the activation of an inflammatory mediator TNF-α (“Tumor Necrosis factor”) and its downstream signaling molecules [185]. In 2008, Nakamura *et al.* hypothesized that complanine binds to the phospholipid binding site of PKC, and later confirmed this to be true [186]. In addition to verification of complanine-induced activation of the PKC inflammation cascade, Nakamura *et al.* (2010) also showed that *E.* *complanata* produces chiral compounds similar to complanine, called neocomplanines A and B, which seem to enhance PKC mediated inflammation. Experimental verification of the inflammation inducing properties of complanine were shown with bioactivity assays and inflammation profiling in mouse footpads, where injection of complanine consistently induced rapid swelling and irritation [177,186].

In humans, skin puncture and subsequent injection of complanine from the chaetae of amphinomids can produce serious inflammation or dermatitis, in addition to burning pain, erythema, numbness and itching that may last several hours [153]. Although toxicity of complanine seems not to show any long lasting detrimental effects, aside from those stated above it still remains an effective deterrent to ward of potential threats. Some of the remaining questions about this toxin that deserve further study include identification of the producing tissue, its ecological role in the wide range of genera and species in Amphinomida, and potentially identification of additional compounds, both non-peptidic and proteinaceous compounds. These questions could be approached with a wide variety of investigatory methods such as deep NGS sequencing of surrounding tissue related to chaetae, proteomic analyses, and additional bioactivity assays on ecologically relevant species such as predator/prey associated species to Amphinomidae.

#### 3.1.3. Earthworms (*Eisenia*)

Annelida is comprised of two major groups: Polychaeta and Clitellata (oligochaetes and leeches) [145]. Approximately 8000 species of oligochaete have been described to date, with about half of these species more commonly referred to as “earthworms” (Lumbricidae) [187]. Despite that no known earthworm species is considered venomous, numerous bioactive secretions are known to be produced by a multitude of species within this group [188,189,190]. Medicinal use of various earthworm-derived secretions has been documented for centuries. People around the world have capitalized on earthworm “toxins” by incorporating their use into cultural practices (e.g., by ingestion or external application) [189,191]. One example, found in Chinese traditional medicine is the use of an ointment prepared from dried innards of the earthworm *Lumbricus rubellus*, called “Di Long” (literally meaning “earth dragon”). It is used to treat ailments including convulsions, arthritis and blepharoptosis (eye drooping).

The importance of nutrient recycling and the general improvement of soil ecosystems driven by the mechanical processes of earthworms cannot be overstated. Vermicomposting, the physical and biochemical degradation of organic matter by earthworms, in association with microorganisms, is a common practice worldwide [192]. This stems in part from the ability of earthworms to boost soil fertility via microbial dense fecal matter (“casts”) deposits [193]. Two closely related species *Eisenia andrei* and in particular *Eisenia fetida* (with many common names including “redworm”, “tiger worm” and “brandling worm”) are highly adapted to and heavily utilized in vermicomposting [192,194]. Due to the suitability of *Eisenia* spp. in composting many of their secreted biomolecules have been investigated. Furthermore, not only are earthworms excellent reservoirs of novel biomolecules, they are also extensively used in ecotoxicology, physiology, biochemical and genetic studies [194], while also having economic value in the fish bait industry [192].

Soil composts are a material rich in nutrients such as proteins and carbohydrates. Earthworms must therefore compete against and evade infection by potentially pathogenic microorganisms [195]. Innate immunity in earthworms and other invertebrates such as molluscs and arthropods, is mediated by their coelomic fluids (CF) and by the activities of specialized secretory cells like coelomocytes and chloragocytes, in addition to humoral (related to body fluids) proteins found in the CF [196,197]. Earthworms have evolved a host of broadly acting defensive antimicrobial proteins/peptides that mediate their immunity [198]. The CF of earthworms, in particular *E. fetida*, has been studied extensively, and results show that they possess a wide range of diversely acting biomolecules including hemolytic hemaggulation, pore-forming, hemolytic, protease, cytotoxic, vasodepressor as well as antimicrobial proteins and peptides [187,189,191,196]. Furthermore, *E. fetida* has an innovative and intriguing defense mechanism: It forcefully expels its CF when attacked or threatened. Coincidently the name given to the pungent expulsion, “fetid” fluid, literally translates from Latin to foul smelling.

Research has led to medical innovations as novel pharmaceuticals have been developed based on distinct proteases and fibrinolytic enzymes isolated from different earthworm species [189,191]. One of the more intriguing classes of secretory proteins identified is that of the lysenin family of earthworm toxins. First isolated in *E. fetida* [199] lysenin is a 33 kDa pore-forming toxin (300 amino acids) that interacts specifically with the major phospholipid sphingomyelin [200] present in high amounts in mammalian cells. Lysenin is known to have high sequence similarity to other lysenin-like proteins (lysenin-like-1, -2, -3) [197,201], while it has also been shown to share structural similarity to many other distantly related pore-forming toxins present in diverse prokaryotes and eukaryotes [200,202]. Lysenin’s specific affinity for sphingomyelin containing membranes, which are present in mammalian and vertebrate cells (but absent in the majority of invertebrates), confers potent toxicity on lysenin. It lyses cells by oligomerization and subsequent ion-channel pore formation [188,201]. Analyses of lysenin activity have shown its ability to lyse many different vertebrate cell lines including sheep erythrocytes [201]. It is also able to induce severe contraction of smooth muscle in rat aorta [195]. Interestingly, with lysenin restricted in its specificity to sphingomyelin, which is a phospholipid group largely absent in invertebrates (absent in Lophotrochozoa with the exception of some mollusc species) [188], this toxin may represent a defensive adaptation of *E. fetida* against vertebrate predators.

Earthworms find themselves in a peculiar position in terms of the active use of toxins, and how they should be viewed in comparison to venomous taxa across the animals. Traditionally defined, venomous organisms produce, store and deliver venom via an inflicted wound to target organisms [4]. However, recent reconsiderations may warrant a broadening of the definition of venoms and venomous organisms [67]. In accordance with [67], considering its vertebrate specific pore-forming toxins and its innovative defensive strategy, *E. fetida* and other species like it might be better distinguished as “autoaglandular-toxungens”. Autoaglandular-toxungens as defined by [67] are capable of synthesizing toxins, possess a delivery method, but lack the glands to store toxins or the capability to inflict a wound. Indeed, an autoaglandular-toxungen classification seems to be very rare, with [67] actually being unaware of any such organisms existing. In light of the multitude of hitherto unidentified toxins, along with the rich diversity of neglected earthworm species, not to mention the seemingly rare autoaglandular-toxungen status of *E. fetida*, should hopefully stimulate much more research into these fascinating creatures.

#### 3.1.4. Bloodworms (Glyceridae)

**Figure 8:**
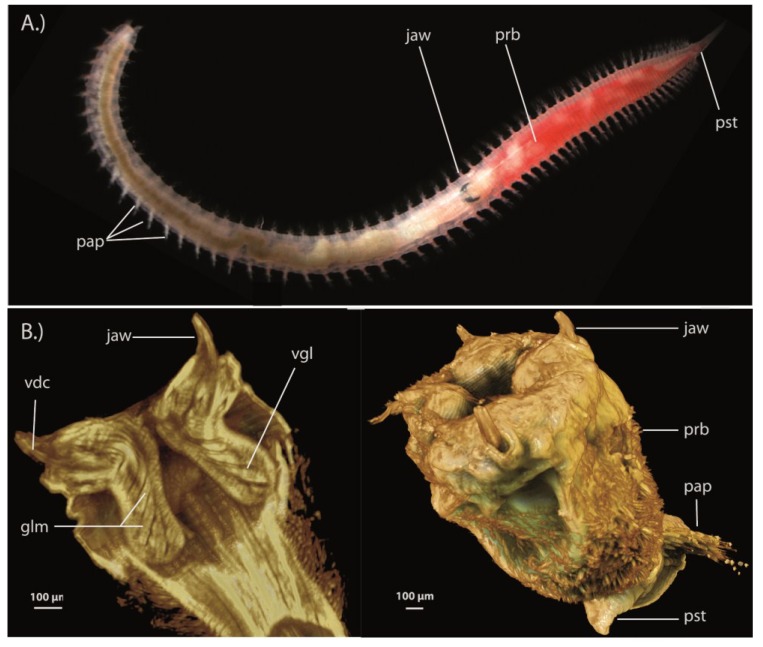
(**A**) Anatomy and general morphology of a *Glycera* bloodworm; (**B**) Rendered micro-CT picture of longitudinal section through everted four jaws and proboscis (**left**), and outer view from above (**right**) of *Glycera tesselata*. glm = muscles associated with the venom glands; pap = parapodium; prb = proboscis; pst = prostomium; vdc = venom duct; vgl = venom gland.

*Glycera* is a genus of marine polychaetes, referred to as “bloodworms” owing to the sometimes-reddish hue attributable to the hemoglobin in their body fluids. Bloodworms, members of the order Phyllodocida, suborder Glyceroformia (*sensu* Grube, 1850) currently comprise 42 described species [203], with most species classified in the genus *Glycera*. All of these share a remarkably homogeneous morphology. Distributed around the globe, they exist mostly as burrowers in muddy bottoms of intertidal zones, but can also be epibenthic on rock substrata and even occur at abyssal depths [178]. Their most striking morphological feature is the presence of a strong muscular eversible pharynx, equipped with four tough, abrasion resistant jaws to deliver venom (see Figure 8). Bloodworms sense potential prey by subtle changes in hydrostatic pressure, and once detected quickly shoot out their proboscis to grasp prey [204,205]. Each jaw tip has ventral side pore-openings connected via a venom duct to a single secretory gland [178,206] (Figure 8B) while the entire jaw is composed of a melanin-like network enriched and strengthened by low amounts of a copper-based biomineral “atacamite” [207].

The majority of bloodworm species appear to be carnivorous, which on the face of their venom delivery apparatus does not seem surprising. Empirical evidence supporting this position stems from the variety of macroscopic invertebrate prey identified via gut content analysis such as polychaetes, molluscs and crustaceans [208,209], in addition to consumption of enteropneusts observed in predation experiments [210]. Conversely, a detritivorous nature of *Glycera* has been suggested [151] following observations of *Glycera* *dibranchiata* becoming compacted with sediment after a period of captivity [147]. However, irrespective of the significance of any sediment ingestion in bloodworms the numerous active toxins observed to be present in their venoms as well as the diversity of toxin gene homologs expressed in their venom glands indicate that they are effective predators as well.

Despite the biological activity of *Glycera* venom being known for some time, until recently the only data on their venom has been protein data. Early investigations of venom from *Glycera tridactyla* (formerly *G. convoluta*) and *Glycera dibranchiata* provided the first glimpse of not only enzymatic but also neurotoxic activity [211,212,213]. Crude venom injected into crustaceans, a natural prey group of *Glycera* [147], induced toxic effects such as cardiac arrest, paralysis, convulsions and ultimately death [211,212,213]. Human envenomation by bloodworms is uncommon but can occur. Symptoms of envenomation are not known to be life threatening, but can include severe dermatitis and local inflammation of the affected area [205]. Testimony from bloodworm bait handlers suggests that they may come to suffer from increased allergic sensitivity following repeated bloodworm bites.

Analysis of protein data derived from the venom cocktail of *Glycera tridactyla* led to the discovery of a high molecular weight glycoprotein, dubbed glycerotoxin (GLTx). GLTx is a 320 kDa neurotoxin that reversibly stimulates the release of neurotransmitters by selectively binding to pre-synaptic Ca_v_2.2+ ion channels (*N*-type Ca^2+^ channel) [204,205]. The action of GLTx is similar to that of the black widow derived vertebrate specific α-latrotoxin in that GLTx stimulates neurotransmitter exocytosis, but differs by actively preventing depletion of neurotransmitter transporting vesicles [214], thus making it an excellent research tool [215]. In addition to the identification of low molecular weight components, additional studies then further characterized [211,212,213] the biological activity of crude *Glycera tridactyla* fractionated venom and showed that independent of GLTx, this venom had protease and phospholipase activities, while also conferring varying levels of lethal toxicity to crustaceans. Moreover, similarities of another unidentified *Glycera* toxin to that of spider α–latrotoxin which forms pores in plasma membranes has been reported [205,211]. Despite the valuable insights gleaned from those pioneering studies, studies on bloodworm venom have remained exclusively tied to more or less restricted investigations of isolated protein fractions.

**Figure 9:**
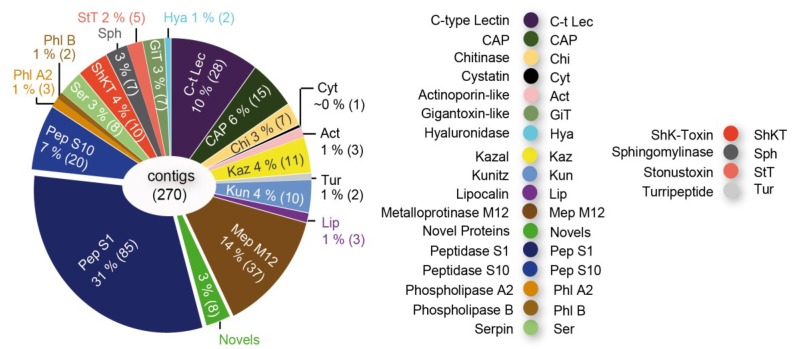
Transcriptome profile of toxin genes expressed in the venom glands of *Glycera dibranchiata*. Pie chart shows the contig diversity of the 20 different toxins expressed in the most deeply sequenced species *Glycera dibranchiata*. Relative contig diversity is expressed as percentages followed by total numbers of contigs in parentheses. See [155] for full details.

Application of Next-gen transcriptomic analyses to explore in detail the composition of venom cocktails is leading to a much deeper and thorough understanding of toxin family acquisition and evolution across venomous taxa [216,217,218]. One of the most recent applications of NGS technology in the study of venom resides with the bloodworms themselves. Von Reumont *et al.* (2014) [155] applied Illumina based deep sequencing to a selection of venom gland transcriptome libraries from three *Glycera* species (*G. dibranchiata*, *G. tridactyla* and *G. fallax*), and one body tissue library (*G. tridactyla*). The authors reported a surprisingly diverse repertoire of previously identified convergently recruited toxin families in addition to 12 putative *Glycera* specific toxin genes. In total 20 convergently recruited toxin families were identified and categorized into five broad functional groups: pore-forming toxins; neurotoxins; protease inhibitors; other enzymes; and CAP domain proteins; see Figure 9. Aside from the functional heterogeneity of the different *Glycera* venom protein genes it is interesting to note bloodworms have convergently recruited toxin families known to be present in disparate and distantly related venomous animals such as scorpaeniform fish, monotremes, gastropod molluscs and cnidarians. Furthermore, this study uncovered candidate toxin families that could help explain many of bloodworm venom effects observed in bioactivity studies. Von Reumont *et al.* (2014) therefore supported *Glycera* being recognized as adept and capable predators of macroscopic prey, but recognized further studies are needed to elucidate specific toxin activity on natural prey groups.

#### 3.1.5. Scale-Worms (Aphroditifomia)

**Figure 10:**
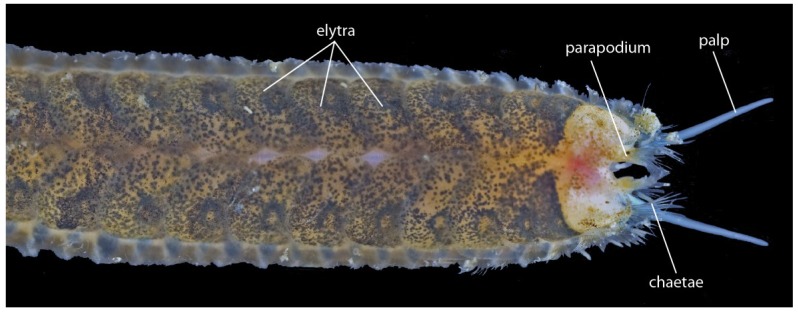
General body morphology of scale-worm polychaetes. Individual shown is an unidentified polynoid species. Copyright for this picture resides with Helena Wiklund, and is reproduced with her permission.

Scale-worms are segmented polychaete worms. Traditionally this group “Aphroditiformia” is recognized to include seven families: Acoetidae, Aphroditidae, Eulepethidae, Pholoidae, Pholoididae, Polynoidae and Sigalionidae (Aphroditiformia), with roughly 1200 species, and 220 genera described to date [178,219]. Morphology of scale-worms varies considerably, particularly the body shape ranging from vermiform to elliptic. Scale-worms possess unique dorsal scales or “elytra”, which represent their most striking morphological apomorphy; See Figure 10 for a representative example of scale-worm morphology. Elytra seem to serve a number of purposes from startling predators, to circulating water and brooding eggs [219]. However, some species such as *Pisione remota* and *Palmyra auriera* have been documented to lack dorsal elytra, despite them being regarded as members of Sigalionidae [154,220,221].

In terms of venom, Aphroditiformia are one of the least well-understood invertebrate groups. Scientific literature focusing on venom use in scale-worms is essentially absent, even in comparison to the other neglected venomous polychaete group *Glycera* that only recently received an in depth molecular investigation [155]. Initial investigations focusing either directly or indirectly on scale-worm venom use revolves predominantly around anatomical investigations of jaw morphology, venom gland presence *vs.* absence or experimental observations of trophic habits of captive or free-living individuals. Generally, scale-worms are large bodied macroscopic animals, with a muscular eversible pharynx equipped with formidable piercing jaws [178]. Presence of jaws is universal in scale-worms, yet distinctly varies between families. For instance, the most basally branching groups Aphroditidae and Eulepethidae reported in Wikland *et al.* [221] and Norlinder *et al.* [219] have species that are known to only possess chitinous “plate-like” jaws [222]; nevertheless some authors regard Aphroditidae as active slow-moving carnivores [147]. However, Wolf [154] did not observe the presence of jaw associated venom glands in Eulepethidae and Aphroditidae.

Evidence for the presence of venom in scale-worms is indirect, and based on the reported presence of jaw-associated glands. One anatomical study in a then novel genus *Metaxypsamma* of the family Sigalionidae by Wolf [154] (now reassigned to Pholoidae) described a scale-worm possessing an eversible muscular proboscis with two pairs of chitinous piercing jaws associated with venom glands adhering to ventrolateral plates of each jaw. Additional insight into scale-worms by Wolf [154] further demonstrated presence of piercing jaws and associated venom glands for three sigalionid species (*Sthenelais* sp., *Psammolyce ctenidophora*, and *Ehlersileanira incisa*). Fauchald and Jumars (1979) [147] conducted one of the most comprehensive reviews into the dietary habits of polychaetes. Trophic habits of the scale-worm family Polynoidae were examined, leading the authors to conclude polynoids to be active, non-tubicolous (non-tube building) motile carnivores. The authors go on to cite a large number of feeding studies for polynoids, collating previous observations of predatory feeding on small crustaceans, echinoderms, gastropods, sponges, hydroids and other polychaetes. Additionally, in the remaining scale-worm families, the species *P. lupines* of the then family Polyodontidae (now accepted as Acoetidae), *Lepidasthenia* sp. (Polynoidae) and *Pholoides bermudensis* of the family Pholoididae (now accepted as Sigalionidae) were shown to also possess very similar jaws and associated venom glands to that of *Metaxypsamma uebelackerae* (Pholoidae) [154]. Similarly, Wolf described another scale-worm, then belonging to Pisionida (since reassigned [219]; see above) as also having piercing jaws and venom glands connected via venom ducts to the tip of each jaw. Peculiarly, in comparison to the known venom pore openings observed in *Glycera* jaws, Wolf [154] did not observe distinct pore openings on the jaws of scale-worms investigated, despite venom canals extending to the very tip of the jaw. To date, this discrepancy of venom gland presence *versus* no discernable pore opening remains perplexing; however it seems likely that venom secreted in these glands has the ability to be expelled into prey following conclusions of the predatory feeding behavior of sigalionids supported by Fauchald and Jumars [147].

Considering the tiny amount of existing knowledge regarding potentially venomous scale-worms, future research of scale-worm venom should likely uncover venomous representatives. Investigations into this group of polychaetes has already begun as our lab is now using venom gland transcriptomics to analyze two species of scale-worm: a polynoid (*Harmethoe imbricata*) and sigalionid (*Sthenelais boa*). Preliminary data show that similar to *Glycera*, scale-worms seem to possess a diverse set of putative toxin homologs [155]. However, these results require future validation.

### 3.2. Flatworms (Platyhelminthes)

About 30,000 species of platyhelminths or flatworms are known [111]. Although flatworms have not evolved anatomically distinct venom glands, several parasitic and free-living species are able to secrete bioactive compounds, some with demonstrated or suspected roles in defense or predation. However, only a handful of species have been studied, resulting in the identification of just a few bioactive compounds.

Toxins are used by a diversity of flatworm species both for defense and for predation. Tetrodotoxin (TTX) is present in different tissues and eggs of several species of flatworm, and it is suspected to function as an anti-predator toxin [223,224]. Interestingly, an unidentified species of planocerid polyclad seems to use TTX, which occurs in a high concentration in its pharynx, to subdue and kill prey [225]. How the worm delivers TTX to the prey is unknown, despite the claim by Williams (2010) [226] that the worm injects the TTX.

Interestingly, a number of benthic and pelagic species of typhloplanid rhabdocoels are able to kill insect and crustacean prey using fast acting neurotoxins of unknown nature [227,228]. Benthic species of *Mesostoma* secrete mucus and a neurotoxin that is able to subdue and kill prey without the need for direct contact between predator and prey [227]. Work on a species of catenulid platyhelminth was similarly taken to suggest the possibility that it is able to secrete toxins able to kill prey [229], but the slow effect (over several days) of the putative catenulid toxin leaves it doubtful whether its primary function is in predation. This interpretation finds support from a detailed study of prey capture and feeding behavior in another *Mesostoma* species [230]. This study found no evidence that the animals employ a secreted neurotoxin, and instead it suggests that the mucus secreted by the worms can function as a mechinal trap of prey, and that dead prey seemingly overcome by the secreted toxin were actually playing dead to avoid detection by the worms.

In striking contrast, pelagic *Mesostoma* species seem to be able to inject a neurotoxin into prey that locally paralyses muscles [228], and which allows the worm to overcome its prey. The envenomation event is extraordinarily rapid, with contact between predator and prey lasting less than 40 milliseconds [228]. Unfortunately it is unknown how the worm manages to deliver its toxin.

Finally, several species of parasitic and free-living platyhelminths express a diversity of secreted peptides, including pore-forming peptides and venom allergen-like proteins [231,232]. The latter are related to venom allergens widely distributed in animal venoms, but the biological roles of these platyhelminth proteins, and whether they are likely to function as predatory or defensive toxins, remains unknown.

### 3.3. Ribbon Worms (Nemertea)

Nemerteans or ribbon worms are mostly carnivorous and mostly marine worms. About 1200 species have so far been described [111]. Nemerteans have an eversible proboscis that can be protruded with great speed. It is used to wrap around and/or pierce the prey, and secreted toxins may assist in subdueing and/or killing the prey. Traditionally nemerteans are classified into three main groups: palaeonemerteans, heteronemerteans, and hoplonemerteans. Only hoplonemerteans possess a proboscis armed with one or several nail-shaped calcarerous stylets that are used to stab and pierce the body wall of prey animals, and which are thought to be used for the injection of venom [233,234]. Hoplonemerteans are therefore also referred to as armed nemerteans (enoplans), with the remaining groups known as unarmed nemerteans (anoplans). Molecular phylogenetic analyses suggest that palaeonemerteans and anoplans are paraphyletic assemblages [235,236] (but see [237]), but because the literature overwhelmingly uses these traditional terms we use them in the present paper as well.

The nemertean proboscis is a cyclindrical body wall invagination surrounded by a fluid filled coelomic cavity known as the rhynchocoel. The proboscis is located dorsal of the digestive system, and runs from an anterior body wall invagination, known as the rhynchodeum, to the posterior end of the rhynchcoel, where it is attached to a retractor muscle in most species. Contraction of the muscles surrounding the rhynchocoel everts the proboscis, while retraction happens by contraction of the retractor muscle. In anoplans the rhynchodeum opens antero-dorsal to the mouth, while in enoplans the mouth and rhynchodeum share an opening to the outside.

**Figure 11:**
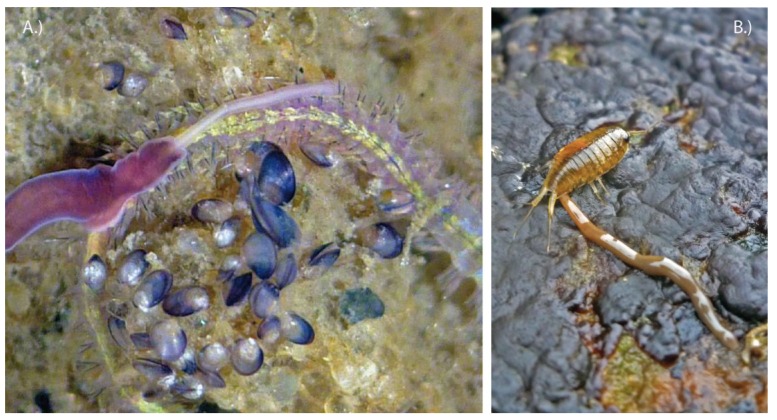
Predating nemerteans. (**A**) Heteronemertean species *Ramphogordius sanguineus* feeding on the polychaete *Alitta succinea*; (**B**) Hoplonemertean species *Prosorhochmus nelsoni* feeding on the isopod *Ligia* sp. Copyright of both photos is with Serena Caplins, and are reproduced with her permission.

Nemerteans are effective predators, and they tackle a diversity of invertebrate prey, including polychaetes, molluscs, crustaceans, and insects. Figure 11 shows nemerteans feeding on a polychaete (A) and on *Ligia*, an isopod crustacean (B). Individual species, however, may not be generalists, and may specialize on different prey types [233,238]. Most nemerteans adopt one of two main feeding modes [238]. The most widespread feeding mode (found in all three traditional taxa) is macrophagy, in which large bodied prey (or carrion) is ingested, sometimes with a diameter several times larger than the diameter of the nemertean. The second, more specialized, feeding mode is suctorial feeding, which is only found in several groups of hoplonemerteans. These worms feed by sucking the contents of relatively large or hard bodied prey through an opening made in the body wall of the prey, or even by entering the prey’s body completely [233,238].

Nemertean predation is thought to be assisted by the delivery of toxins to the prey by the proboscis, either via the mucus it secretes, or directly via wounds inflicted by the stylet of hoplonemerteans. Secretory cells lining the proboscis produce and secrete a sticky and toxic mucus [231,239,240], but nemerteans do not have morphologically distinguishable venom glands. Toxins are thought to be produced by specialized gland cells. In hoplonemerteans the epithelial lining of the anterior and posterior chambers of the proboscis produce different types of secretions [239] and toxins are thought to be produced and secreted principally in the anterior chamber. These chambers are separated by the stylet apparatus in hopolonemerteans.

The hoplonemertean stylet apparatus may contain a single central stylet, usually between 50–350 μm long, or multiple smaller (less than 15 μm long) stylets. The stylet is used to stab and puncture prey, and hoplonemerteans frequently stab their victims multiple times. Reserve stylet sacs surround the central stylet, and can replace the stylet if its gets damaged or lost. The stylet apparatus is anchored in a muscular diaphragm, through which runs a narrow canal that connects the anterior and posterior proboscis chambers. This canal allows mixing of the distinctive secretions produced in the proboscis chambers.

Hetero- and palaeonemerteans lack proboscis stylets. However, their proboscis epithelial cells contain rhabdoids, small rod-shaped secretory bodies. Although some kinds of rhabdoids are secreted as part of the proboscis mucus [239] it has been suggested [241] that some types of rhabdoids are attached to and protrude from proboscis cells, and may puncture the prey body wall so as to facilitate envenomation [239]. However, there is no empirical evidence in support of this idea.

Although the hunting behavior of ribbon worms has been the subject of numerous studies, it remains unclear whether hoplonemerteans are actually able to directly inject toxin into prey via stylet wounds. However, it seems likely that wounds inflicted by the stylet at least allow toxic mucus to enter the prey. In contrast hetero- and palaeonemerteans seem to envenom their prey by applying toxic mucus to the outside of the prey’s body. It has been noted that prey paralysis is limited to the area of the prey contacted directly by the proboscis of the hoplonemerteans *Paranemertes peregrina* [239]. This suggests that toxins are not only introduced via the wound inflicted by the proboscis stylet. Envenomation of prey is also not necessarily fatal, and paralysis can be temporary, with prey being able to recover if the nemertean loses contact with it.

Nemerteans produce a variety of proteinaceous and non-proteinaceous bioactive compounds, which are thought to act as toxins with roles in both defense and predation [231,240]. TTX is probably the most widespread nemertean toxin that confers protection against predation. It has been found in hetero-, palaeo-, and hoplonemerteans [242,243], and is probably produced by symbiotic bacteria [242,244,245]. A putative defensive function has also been attributed to proteins found in mucus secreted by the body surface of hetero- and palaeonemerteans [246]. Such mucus extracts contain powerful neurotoxic peptides that can cause convulsions, paralysis and death of injected crabs, as well as cytolytic proteins expressed in the worm’s skin [231,240,246,247]. Since these protein neurotoxins have a strong and specific toxicity to crustaceans, a common nemertean prey, they may also, or perhaps chiefly, play an important role in predation. However, Kem (1985) [240] doubted this offensive role of heteronemertean toxins, presumably because it is unclear how the worm’s unarmed proboscis manages to deliver the toxins inside the prey. Since field studies may offer contradicting accounts about the paralytic power of specific species [241], more work is clearly needed to determine to what extent and how hetero- and palaeonemerteans use their toxins to subdue prey.

Notably, the only heteronemertean toxins characterized and purified to date derive from two large bodied species, *Cerebratulus lacteus*, and *Parborlasia corrugatus* [231]. However, the expression of such putative defensive compounds in the skin mucus of species such as *C. lacteus* does not provide a universally effective defense against all types of predators. While some potential predators of nemerteans strongly reject nemertean prey, a range of vertebrates and invertebrates are known to prey upon nemerteans [238,248].

In contrast to the hetero- and palaeonemerteans studied to date, hoplonemerteans do not seem to express cytolytic and neurotoxic proteins. Instead they express non-proteinaceous compounds, such as the alkaloid neurotoxin anabaseine, which is concentrated in particular in the proboscis, and a tetrapyridyl called nemertelline [231]. The best understood toxin, anabaseine, can cause paralysis of nemertean prey such as polychaetes and crustaceans, and appears to stimulate a wide variety of animal nicotinic acetylcholine receptors [231,247].

Stricker (1985) [249] devised an evolutionary scenario for the origin of the specialized stylet apparatus of hoplonemerteans. In broad outline it starts with an ancestor with toxin secreting cells in its proboscis epithelium. This was followed by the evolution of rhabdoids that could puncture a prey’s body wall, and which became increasingly concentrated in the mid region of the proboscis, while the toxin secreting cells became mostly restricted to the anterior proboscis chamber. Increasing calcification of the rhabdoids would then lead to the situation observed in polystiliferan hoplonemerteans, which possess multiple smaller calcified stylets, and finally to the single stylet system found in monostiliferan hoplonemerteans. The intermediary stages in this scenario are exemplified by the rhabdoid systems of hetero- and palaeonemerteans. Stricker emphasized the early evolution of rhabdoids that could inflict wounds on prey because he thought that was needed for the effective delivery of toxins. However, any penetrating role that rhabdoids may have in envenomation remains unproven. Yet, this scenario is at least in broad agreement with our current understanding of nemertean phylogeny [235,236].

Nemerteans are a promising and neglected group of venomous organisms in need of in depth study to answer the many interesting questions that remain. These including the role of toxins in catching prey (in particular in hetero- and palaeonemerteans), the mechanisms used by nemerteans to deliver their toxins, especially the relative roles of delivering toxins to the outside and inside of the prey’s body, as well as a better understanding of the diversity of proteinaceous and non-proteinaceous toxins secreted by the proboscis epithelium as well as other tissues.

### 3.4. Mollusca

Although cone snails are among the most studied and best understood of all venomous animals, the 700 or so described species represent only a fraction of the total diversity of venomous molluscs, which exceeds over 10,000 species [250]. The vast majority of venomous molluscs, all of which are marine, were traditionally contained within three families of the superfamily Conoidea: cone snails (Conidae), auger snails (Terebridae), and especially turrids (Turridae), a group which comprises over 90% of venomous conoideans. But venomous gastropod species also occur outside Conoidea in the tonnoidean families Rannelidae, Muricidae, and Cassidae [251], as well as in coleoid cephalopods [4,252].

The biology and evolution of the venom apparatus and venom toxins of cone snails have been intensely studied [250]. In contrast, the other conoidean groups have been almost completely neglected in venomics studies, but recent studies show that their detailed study would be extremely beneficial. Turrids are a very diverse group of specialist predatory marine snails [253]. The benchmark molecular phylogeny of Conoidea produced by Puillandre *et al.* [254] shows that turrids form a paraphyletic grade in which the cone snails and auger snails are placed as two distantly related clades. Studying turrids is therefore important for understanding the evolution of the venom systems of cone and auger snails as well.

Many turrids are small snails (on average between 3–50 mm), which may in part explain their relative neglect in venomics studies. Turrid taxa for which the venom has been studied [255,256,257,258,259,260] are placed in three of the 13 families (Clavatulidae, Turridae, and Pseudomelatomidae), leaving a vast unexplored phylogenetic panorama. Turrid venom has been shown to have a variety of proteolytic, hemolytic, cytotoxic and neurotoxic effects [256], and turrid toxins represent a mixture of mostly cystein-rich peptides, most of which are strikingly different from conotoxins [257,258,260]. Only one group of turrid toxins shows conspicuous similarity to conotoxins of the I_2_ family [257]. Since the turrid species for which venom toxins have been studied are located in a different major clade than the one that contains cone snails [254] these results indicate that many turrid toxins may have radiated independently of conotoxins.

Auger snails (350–400 described species) form a clade in the phylogeny of Puillandre *et al.* [254] which is the sister group to a clade Turridae *sensu stricto* that contains the genus *Turris*. A remarkable aspect of the evolution of terebrids—and in striking contrast to cone snails—is the frequent loss of the venom apparatus [261,262,263,264]. Similarly, hypodermic radular teeth have evolved at least three times in terebrids, and independently of those in cone snails [264]. Castelin *et al.* [264] note that foregut anatomy of auger snails is at least as diverse as that found in cone snails, and that its evolution is correlated with dietary specializations. At the present, however, this remains an untested idea, as the diets of most terebrids are unknown.

Terebrid venom toxins are referred to as teretoxins (previously referred to as augertoxins). Terebrid venom has been analysed in only four species, *Terebra argus*, *T. consobrina*, *T. subulata*, and *Hastula hectica* [263,265,266]. Teretoxins seem to target acetylcholine receptors [266]. The mature sequences of some teretoxins are similar to, but larger than, conotoxins [263], but the signal peptides are entirely distinct from those of conotoxins. The absence of homology between some teretoxins and conotoxins conforms with the disjunct phylogenetic positions of cone and auger snails in the conoidean phylogeny. However, given the vastly underexplored venomics territory of auger snails and turrids much more research is needed to draw robust conclusions about the composition, activities, and interrelationships of these conoidean toxins.

Molluscan venoms, however, are not limited to conoideans. Species in the gastropod families Ranellidae (tritons) and Cassidae (helmet shells), as well is in Muricidae (rock snails or purple dye snails), are also thought to use toxic secretions to subdue prey [251]. The large salivary glands of ranellids and crassiids can produce sulphuric acid and tetramine, but also produce peptide toxins that can paralyse and kill prey [251,267]. Shiomi *et al.* [267] purified three lethal and hemolytic peptide toxins from the ranellid *Monoplex echo*, and called them echotoxins. Subsequently Kawashima *et al.* [268] elucidated the primary structure of one of these echotoxins, and found it to be similar to actinoporins, which are hemolytic pore-forming toxins initially described from sea anemones, but now also found to be expressed in the venom glands of platypus and bloodworms [155]. Finally the more distantly related muricid gastropods are also able to produce toxic secretions (including acid, choline esters, and enzymes) with their accessory boring organ, accessory salivary glands, and hypobranchial gland [251,269]. These secretions are used to bore holes through the shells of prey, but may also interfere with the prey’s physiology and assist in prey capture. The hypobranchial gland secretions turn purple when exposed to light and air, and muricid prey often show purple discolorations. Several other gastropod families are also able to produce purple dye, but its actual role in subduing prey remains mysterious.

Molluscan predatory venoms are also present outside Gastropoda in coleoid cephalopods. The recent studies by Undheim *et al.*, Fry *et al.* and Ruder *et al.* [4,252,270] have shed light on the bioactivities and identity of proteinaceous venom toxins expressed in the anterior and particularly the posterior salivary glands of several species of squid, cuttlefish and octopus. These molluscs express a diversity of proteins and peptides that are thought to play important roles in subduing and digesting prey. Among the toxins for which the glands express precursors are enzymes including chitinase, hyaluronidase, peptidase S1 and phospholipase A_2_ which are commonly expressed in animal venoms, as well as possibly lineage-specific peptides. These results show that coleoid cephalopods are a promising area for further research into endogenously produced venom toxins.

Finally, it is known that blue-ringed octopuses (genus *Hapalochlaena*) harbor tetrodotoxin (TTX) in their tissues, especially in the cells lining the secretory tubules of the posterior salivary glands [271]. This suggests that the animals are able to use TTX to help overcome prey, using it in addition to the proteins and peptides it produces in its salivary glands. However, TTX can also play a role in defense. Bites of *Hapalochlaena* species can be deadly to humans and cause symptoms corresponding to TTX poisoning [272]. This suggests that TTX can be injected into prey by biting, but ingestion of blue-ringed octopus can be equally fatal [273].

## 4. Echinoderms (Sea Stars, Sea Urchins)

Echinoderms are a clade of marine invertebrate deuterostomes that includes some well-known subgroups such as sea urchins, sea stars, sea cucumbers, sea lilies and sand dollars. This group is characterized by possession of a calcium carbonate enriched skeleton, five-fold radial symmetry and usually many spines/thorns as seen in the easily recognizable sea stars. Echinoderms are distributed throughout all seas, inhabiting shallow to deep benthic environments with a distribution ranging from tropical seas to artic zones. The current estimate of echinoderm species is approximately ~20,500 of which there are thought to be around ~13,000 fossil species [111].

Of the members of Echinodermata, current knowledge indicates that there are only two groups of interest in relation to the evolution of venom: Asteroidea (sea stars) and Echinoidea (sea urchins) [274]. Numerous poisonous echinoderm species have been identified, for example members of Holothuroidea (sea cucumbers) produce “holothurinogenins” [275,276], which can cause digestive problems or in rare cases death when eaten [277]. The sea star *Acanthaster planci* (“crown-of-thorns sea star”) represents a peculiar case for echinoderms as it seems to be both poisonous and venomous [278,279], while also being the only Asteroidea species known to have evolved venom [280,281]. The sting from *Acanthaster planci* can cause a range of symptoms including intense pain, redness, swelling and protracted vomiting [279,280], while it can also be fatal in rare cases [282]. Finally, of the approximately 850 living species of sea urchins [283] a number of species have been shown to produce and inject potent venom. Sea urchins are omnivorous, feeding upon organisms such as algae, molluscs and foraminifera [274], yet they contribute to the overwhelming majority of human-echinoderm envenomation cases. Reports of symptoms following envenomation include extreme pain, reddish swelling, and in severe cases temporary paralysis and irregular pulse are known [277]. There are two basic types of venomous appendages used for the purpose of defense in sea urchins and other echinoderms: spines and small grasping “pedicellariae” [284].

**Figure 12:**
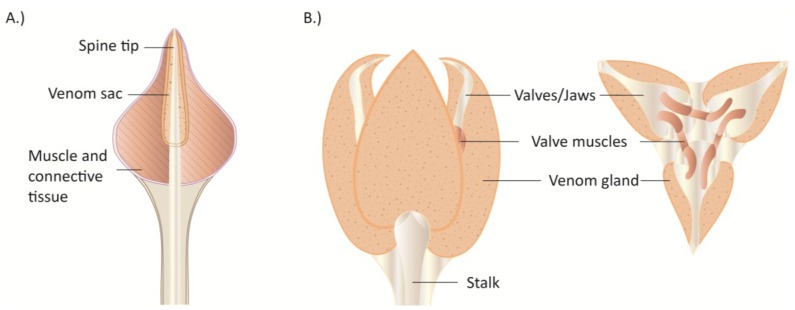
Venom appendages of sea urchins (Echinoidea). (**A**) A secondary aboral spine of *Asthenosoma vaium,* showing spine tip and associated venom gland (sac) and muscle tissue; (**B**) Typical venomous globiferous pedicellaria. Pedicellaria shown is fanged with external venom glands on the valves. Distinct from “Fistulate” globiferous pedicellaria (internally located venom glands) [284]. Figure redrawn and modified from Halstead [274].

Sea urchin spines are highly variable between different groups, and can differ not only in size but also function for a given species. The dorsal venom- and non-venom associated spines function primarily in defense. Sea urchin venom associated spines have a single large venom gland enclosing the point of the spine tip [274]; see Figure 12A. In addition, interspersed among the primary spines sea urchins have smaller modified venom delivering pedicellariae. These appendages are pincer-like structures capable of capturing and injecting venom into prey. However, they chiefly function in defense against larger predators [285]. There are four types of pedicellariae in Echinoidea, but all have the basic structure comprising two parts: a head armed with between 2–5 calcareous valves or “jaws”, and a stalk that supports the head [284]. At least two types of pedicellariae, globiferous (see Figure 12B) and ophicephalous, are known to house either internal or external venom glands, and these are widespread in sea urchins. Interestingly, both the smallest and largest types of pedicellariae (triphyllous and tridentate respectively) have not been confirmed to deliver active venom. The venom glands typically lie at the base of each pedicellaria valve and are connected to the valve tips via venom ducts [284,285]. Coppard *et al.* (2010) detected an evolutionary trend through the Mesozoic and Tertiary towards pedicellaria that are able to deliver venom increasingly effectively via puncture wounds. Sea stars (Asteroidea) also have large primary spines and similar appendages to pedicellariae, however, these are typically bivalved and seem to have originated independently to echinoid pedicellariae; see Coppard *et al.* [284].

Histological evidence suggests that sea star toxins are produced by glandular tissue of their spines [274], which can be stripped off and remain in a wound following spine penetration [279]. Early investigations of crude venom extracted from the crown-of-thorns sea star spines (*Acanthaster planci*) revealed a number of biological activities such as hemolytic activity, phospholipase activity, anticoagulant activity, histamine-releasing activity, capillary permeability-increasing, hemorrhagic and myonecrotic activities; see [280,286]. Currently evidence suggests that there are at least three classes of proteins/peptides that comprise active toxin components of *A. planci* venom: 2 mouse lethal factors “plancitoxin”-I and II [280], phospholipases A_2_ (AP-PLA_2_-I and II) [287,288], and an anticoagulant factor “plancinin” [289]. Plancitoxins are an interesting toxin class. Firstly, they are known to share conserved sequence identity to mammalian deoxyribonuclease II (DNase II) enzymes [281]. Secondly, they seem to be the first identified DNase II hepatotoxin (liver damaging toxin) [280]. And lastly they act by inducing chromosomal DNA fragmentation via caspase-independent apoptosis [290]. Lee *et al.* [291] recently demonstrated the potent hemolytic activity of both crude and lyophilized *A. planci* venom, while Sciani *et al.* [292] observed cysteine peptidase activity in the spine extract of another sea star species *Echinometra lucunter*.

Pioneering toxicology experiments on the activity of crude venom from a variety of sea urchins showed early on the potent nature of Echinoidea venom. Uexkull (1899) first showed that venom extracted from *Sphaerechinus granularis* pedicellariae proved lethal to marine snails and eels, while it could also stop a frog’s heart and induce convulsions following venom injection into its spinal cord. Fujiwara (1935) tested the venom of globiferous pedicellariae of *Toxopneustes pileolus* and showed similar results as found for the venom of *S. granularis* when it was injected into mice abdomens; see Halstead [274] and Kuwabara [293] for additional early investigations of sea urchin venom. Much of what is known about the venom of Echinoidea comes from investigations of a small sample of venomous sea urchins, in particular *T. pileolus* (flower urchin)*.* Kimura *et al.* (1980) [294] characterized the action of *T. pileolus* venom and further showed it could induce muscle contraction and histamine release in isolated smooth muscle [295]. The protein component of *T. pileolus* venom that causes contraction of smooth muscle was subsequently isolated and dubbed Contractin-A [295]. A second toxin, an 83 AA cytochrome b-like heme protein called “Peditoxin” was then characterized from *T. pileolus* and was shown to cause anaphylactic shock and death in experimental animals, both vertebrates and invertebrates [293]. Nakagawa *et al.* [296] identified a novel lectin in *T. pileolus*, while another component was observed to inhibit Ca^2+^ uptake in nerve endings [297]. Although echinoderms have been investigated for their venom for some time, there still remains a large gap in our knowledge, specifically the identification of toxin components across all major venomous taxa. In addition, venomous groups are yet to be sequenced with any modern NGS technology, as reflected by the lack of molecular data on echinoderm toxins available in public sequence repositories.

## 5. Methodological and Future Prospects

### 5.1. Separating Fact from Fiction

Venoms and venomous species have long attracted attention and fascinated people of all kinds. In this review we have discussed venomous and putatively venomous species some of which have been assumed to secrete specific toxins already for hundreds of years. Conclusions that species are indeed venomous are often based on detailed and careful observations of the biology of those taxa, even though evidence about the existence and nature of venoms reported in older papers often remained tentative because the tools to identify toxins were not yet established. The development of highly sensitive techniques to detect venom toxins and their transcripts, as well as their bioactivities have now ushered in a new era for comparative venomics. However, one potential drawback of the current fascination with venoms is that discussions of venomous species by lay people may be exaggerated or not really be based on carefully obtained data. One example are the camel spiders or wind scorpions, which are still often reported to have a strong venom, despite the existence of only one publication that describes venomous effects for one species (see discussion above).

Conversely, astonishingly precise observations given in online forums made by non-scientists have described envenomation effects for example for fly larvae, which are vital for directing further, scientific investigations into these neglected taxa. Interestingly, the recent advances in molecular sequencing techniques allow us now to very easily test for expressed putative toxins in specific tissues, to complement earlier proteomic work that mostly covered only single proteins.

### 5.2. Book of Venom Revelation—-omics Technology as a Game Changer

**Figure 13:**
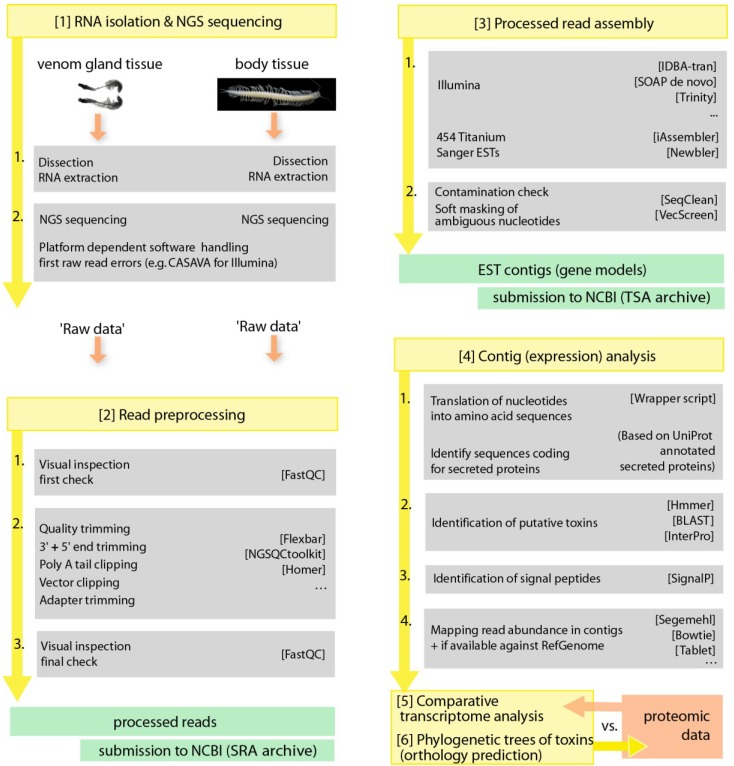
Flowchart of transcriptomic analyses using NGS data to identify putative toxin proteins. Software used to conduct analyses is indicated in square brackets; please keep in mind that these are examples and not an exhaustive presentation of possible software.

Recent technological developments enable us to describe expressed genes in a tissue in a very automatized and cheap way. Pipelines to identify putative venom proteins were recently established [7,155] and the number of publications in which venoms and their components in previously neglected taxa are described grow rapidly. It is important to keep in mind that transcriptome data is used to produce “gene models” or gene-contigs that need supplementary data to confirm if the products they encode truly function as toxins. This annotation is based on a thorough pre-processing of the read data and starts with RNA isolation, see Figure 13. (1) RNA isolation from venom gland tissue and non-venom gland body tissue follows the standard procedures. Procedures may vary according to the extraction kit used, as well as the NGS sequencing platform used; (2) The preprocessing of the reads includes a first visual inspection of those raw-reads using FastQC [298]. Different software tools are available for trimming the data, but these programs may differ in what they can do. Dependent on what sequencing platform is used, adaptors and vector sequences need to be clipped. NGSQCtoolkit [299] is one of the tools that can do all these steps for both 454 Titanium and Illumina data, but see also Flexbar [300] and Homer [301]; (3) The reads can then be assembled using different assembler programs like IDBAtran [302], SOAPdenovo [303], Trinity [304], iAssembler [305], and Newbler (obtainable from Roche via request). Note that different assemblers are not likely to produce identical results. Final contigs or gene models need to be checked for contamination by running SeqClean [306] and VecScreen [307]; (4) Contig or gene expression pattern analysis starts with a translation of the nucleotide sequences into amino acids. To identify venom proteins the search can generally be restricted to transcripts coding for secreted proteins. We use a recently published wrapper script [155] for this that uses BLAST to identify UniProt based secreted proteins. Putative venom proteins can be identified by applying several search strategies, including hidden Markov Models, InterPro scan based domain searches and BLAST procedures. A check for the presence of signal peptides is important to ensure that identified proteins are indeed secreted. To see the abundance or numbers of reads that constitute a contig (or gene model) those reads need to be mapped against the contig using software such as Segemehl [308] or Bowtie [309]. Visualization is possible with tools like Tablet [310]. For further inspections of protein domain arrangements the mapping can be performed against a reference genome if available; (5) The last important step is a comparative analysis of body *vs.* venom gland tissue to identify venom gland specific genes. Ideally these transcriptomic results are then complemented by protein data obtained from proteomic analyses of venom; (6) This step includes also a thorough orthology prediction of putative venom proteins by reconstructing phylogenetic trees for toxins including non-venomous taxa and toxin homolog contigs derived from non-venom gland material, see von Reumont *et al.* [7,155].

The number of annotated and manually curated venom proteins in published databases like UniProt or SwissProt are increasing, see Table 1, and the number of publications in which venoms and their components in previously neglected taxa are described are growing rapidly. The annotations in databases like SwissProt include, besides information derived from BLAST analyses, proteomic and sometimes experimental data on the function of genes, such as data generated by knocking down genes, to predict and identify the function of genes.

The rapid development of the methodological tools may come with the drawback that some errors can be introduced into analyses that try to identify venom toxin genes. Errors can already happen early in transcriptomic analyses, before the reconstruction of contigs. The basis of all transcriptomic work is the assembly of sequenced transcripts, often referred to as reads (see Figure 13). However, assembly is a rather stochastic process and it was shown for secreted proteins from *Glycera* polychaetes that the resulting protein contigs (or gene models) show differences depending on which assembly software was applied. Using the same preprocessing tools before assembly, fewer sequences with regions coding for signalpeptides were discovered with CLC workbench—a commonly used GUI based commercial software package (CLC Genomics Workbench v5.5.x, CLC bio, Aarhus, Denmark), than with IDBA-Tran—a command line based assembly software [155,302].

**Table 1:** Comparison of the total numbers of secreted proteins in UniProt (location: SL 0243) in the timespan of January 2013 to July 2014 is shown to demonstrate the rapid growth of venom related sequences. Numbers of sequence matches for “toxin”, “venom”, “Conotoxin” and “Snake venom” are shown as examples.

SL_0243	Protein sequences	Match “toxins”	Match “venom”	Match conotoxin	Match snake venom
01.2013	53,796	3,638	332	960	130
07.2014	85,146	4,524	528	1,515	225

Interesting is also that so far no direct comparison has been made between older 454 Titanium and more recent Illumina based NGS sequencing technologies in terms of how well they are able to capture the expressed diversity of venom proteins. We suspect, based on our own unpublished results, that specific, or more lowly expressed toxins might be overlooked in less deeply sequenced 454 Titanium platform transcriptomic profiles.

A few recent studies have shown the importance of including non-venom gland related tissue as well as non-venomous species into analyses, if resolving the evolutionary history of specific venom proteins is the goal [3,7,155,311,312]. Many studies still ignore this important methodological aspect. Without a non-venomous taxon the rooting of phylogenetic trees becomes arbitrary and the direction of protein evolution cannot be determined. In this case a non-rooted tree or network is the only option for representing the evolutionary relationships between toxins. If non-venom gland related tissue is not included into analyses all protein variants or paralogs are ignored that might represent ancestral protein variants, impeding any robust phylogenetic conclusions [3,7,155,311,312].

A further area where caution is required is the preference of some authors for Bayesian phylogenetic methods to resolve phylogenetic trees. It seems that this method is sometimes only chosen because of its “effect” of increasing node support values compared to bootstrap analyses of likelihood based approaches. However, this effect can be artificial and not reflect the structure or signal within alignments, which are the basis of phylogenetic reconstructions. This overestimation of node support values by Bayesian statistics is well known to phylogeneticists [313,314].

In general, genome data is still rare despite the existence of genome consortia like i5k [315], in particular for venomous species. To understand the processes that drive venom evolution, more genome based studies are needed. The first studies that investigate venom with genome data indicate that some long established ideas about how venom proteins evolve, for instance via gene duplication [316], might not accurately or comprehensively reflect the possible processes of toxin evolution. The recently published platypus genome, for instance, was the basis for a venom study in which it was shown that gene duplication for this species plays a less important role than previously expected [316]. Furthermore, another genome based study on king cobra venom yielded the observation that proteomic data is incongruent with the transcriptomic data [6]. This interesting observation needs further investigation, in particular in the light of a recent study that showed that snake venom composition is controlled by a variety of transcriptional, translational and posttranslational mechanisms [317]. Based on such new insights derived from genome data in association with expression level data Reyes-Velasco *et al.* [312] proposed a new model for venom protein evolution that differs from the widely accepted model of venom protein evolution that emphasizes the central role of the recruitment of toxin genes to venom gland tissue. Instead the new model proposes that homologs of venom toxin genes are already expressed in a wide diversity of tissues, and that the evolution of venom secreting tissues does not so much require the recruitment of toxin genes as changes in the expression profiles of genes already expressed in the tissue ancestral to the venom gland tissue.

### 5.3. The Unique Value of Incorporating Neglected Taxa into Venomics

Our hope is that this review will inspire researchers to focus more on some of the neglected venomous taxa. Only a broad phylogenetic sampling of venomous taxa will allow us to generate and test general propositions about the biology and evolution of venoms. Some neglected taxa are especially promising for tackling some of the fundamental questions relating to the functions of venoms, for instance the relative roles of venom in defense and predation. For instance, it was recently discovered that previous identifications of conotoxins was heavily biased towards those used in defensive rather than predatory secretions [5]. The evolution of venoms of many better-studied taxa, such as cone snails, scorpions, and spiders is likely influenced by both defensive and predatory needs. However, several neglected venomous groups, such as echinoderms and bloodworms, have likely evolved their venoms mostly or exclusively for defense and predation, respectively. To better understand the contexts in which defensive and predatory toxins function and evolve it would be useful to study these venoms as well. However, an important Achilles Heel for such work in many taxa is the paucity of reliable data about venom function in natural habitats. Understanding how venoms function in natural prey and predators is badly needed, but will be very challenging to obtain, especially for difficult to observe and aquatic taxa, such as remipede crustaceans and polychaetes. However, such basic natural history studies are needed if we wish to understand the natural roles of venoms, rather than just their value as lead compounds for the development of new pharmaceuticals.

## Supplementary Materials

Supplementary materials can be accessed at: http://www.mdpi.com/2072-6651/6/12/3488/s1.
